# TRKB-based signature identifies high-risk squamous cell carcinoma cases and TRKB blockade reprograms tumor and stromal cells toward suppressive phenotypes

**DOI:** 10.1186/s12929-026-01227-0

**Published:** 2026-02-25

**Authors:** Valeria Bartolocci, Alessio Capone, Rosanna Monetta, Erika Di Meo, Silvia Arcano, Carola Valente, Denise Campagna, Massimo Teson, Mara Mancini, Giovanni Di Lella, Damiano Abeni, Luca Fania, Francesca Ricci, Vito Gomes, Giovanni Luca Scaglione, Simona Mastroeni, Eleonora Candi, Elena Dellambra

**Affiliations:** 1 Laboratory of Molecular and Cell Biology, Istituto Dermopatico Dell’Immacolata IDI-IRCCS, Via dei Monti di Creta, 104, 00167 Rome, Italy; 2 Department of Experimental Medicine, University of Rome “Tor Vergata”, Rome, Italy; 3 Biochemistry Laboratory, Istituto Dermopatico Dell’Immacolata IDI-IRCCS, 00167 Rome, Italy; 4 Skin Cancer Center, Istituto Dermopatico Dell’Immacolata IDI-IRCCS, 00167 Rome, Italy; 5 Clinical Epidemiology Unit, Istituto Dermopatico Dell’Immacolata IDI-IRCCS, 00167 Italy Rome,; 6 Anatomic Pathology Department, Istituto Dermopatico Dell’Immacolata IDI-IRCCS, 00167 Rome, Italy; 7 Bioinformatics Unit, Istituto Dermopatico Dell’Immacolata, IDI-IRCCS, 00167 Rome, Italy; 8 National Centre for Disease Prevention and Health Promotion, Italian National Institute of Health, Rome, Italy

**Keywords:** Cutaneous squamous cell carcinoma, TrkB, Cancer-associated fibroblasts, Skin, 3D SCC models

## Abstract

**Background:**

Cutaneous squamous cell carcinoma (cSCC) is a common age-related cancer, with a subset prone to recurrence and metastasis. Currently, no useful diagnostic biomarkers for high-risk cSCC are available. Based on our previous findings, indicating that age-related changes in the neurotrophin receptor tyrosine kinase-2 (TrkB) axis may promote skin tumorigenesis, this study aims to identify novel cSCC biomarkers and therapeutic targets.

**Methods:**

A retrospective analysis was conducted on specimens from patients with in situ or invasive cSCCs using immunohistochemistry to assess the expression of TrkB and specific downstream proteins (i.e., E-cadherin, Yap1, and Notch1). Statistical and machine learning analyses were applied to identify biomarkers that distinguish cSCC subtypes and patient risk groups.

In vitro studies involved treating SCC cells, cancer-associated fibroblasts (CAFs), and three-dimensional (3D) SCC models with the TrkB inhibitor ANA-12. Gene and protein expression were analyzed via RTqPCR, immunoblotting, and immunoassays. Functional assays evaluated cell proliferation, migration, and invasion. Secretomes were profiled using cytokine arrays.

**Results:**

Protein expression levels mainly correlated with cSCC types. Our findings indicated that the *‘TrkB, E-cadherin, Yap1, Notch1’* signature can be a relevant biomarker for both cSCC subtype classification and identification of high-risk cases. Despite the limited sample size, machine learning models demonstrated promising accuracy in differentiating between cSCC classes. Our analysis also highlighted the added value of including stromal markers for classifying high-risk patients.

Furthermore, TrkB blockade suppressed tumorigenic traits in *TP53*-mutant SCC cells, including proliferation, EMT, migration, invasiveness, and disruption of the IL-6/STAT3 signaling loop, while promoting differentiation and senescence through modulation of key players such as p63, Yap1, Notch1, and p21. Data are consistent with a tumor-suppressive effect, thereby promoting tissue homeostasis, especially in physiologically relevant 3D models. Inhibiting TrkB reprogrammed primary CAFs into a less proliferative, migratory, inflammatory, and fibrotic phenotype by simultaneously suppressing key activating pathways, such as β-catenin, Yap1, and Notch1. This aligns with a reduction in their tumor-supportive functions.

**Conclusions:**

Our findings provide a basis for improved high-risk patient stratification by highlighting a TrkB-based signature and generating prototype predictive models. Furthermore, they offer promising therapeutic avenues for developing combined targeted interventions to overcome resistance in high-risk patients.

**Graphical abstract:**

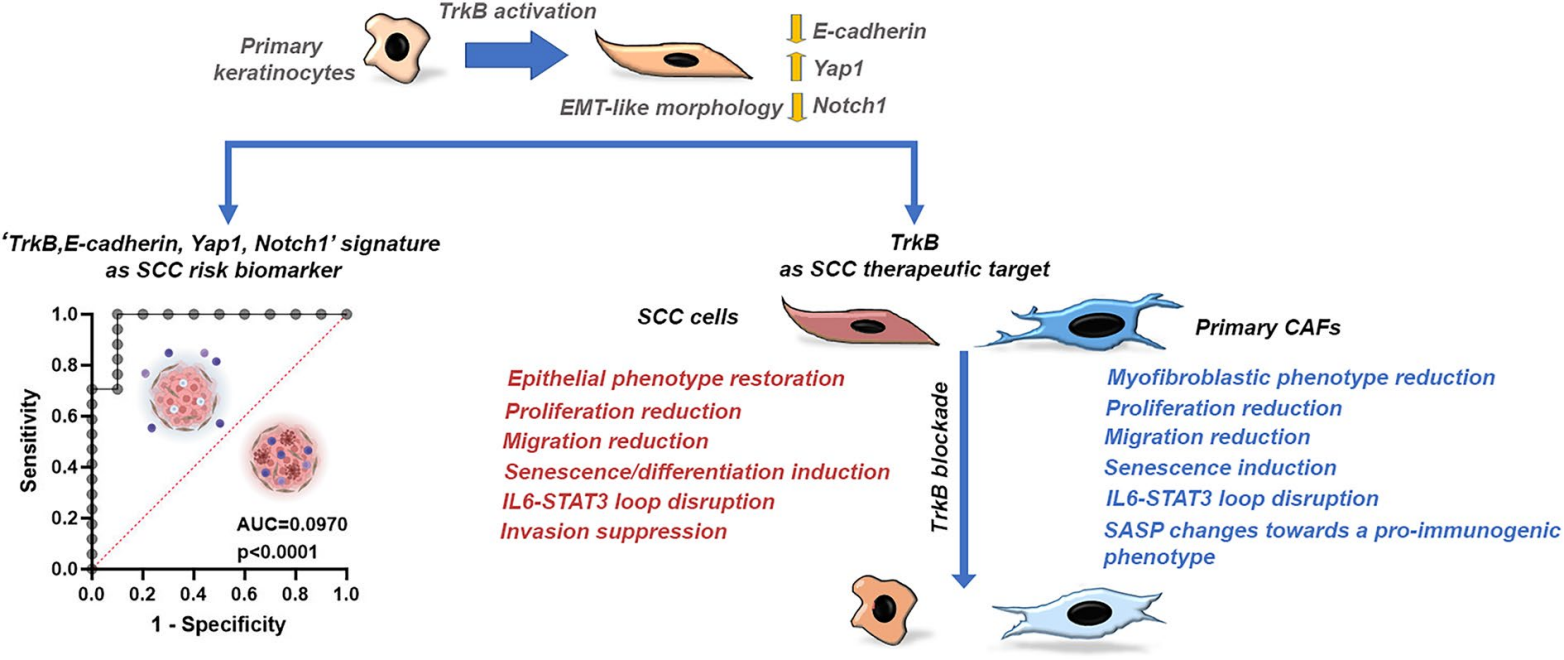

**Supplementary Information:**

The online version contains supplementary material available at 10.1186/s12929-026-01227-0.

## Background

Cutaneous squamous cell carcinoma (cSCC) is the second most common form of non-melanoma skin cancer. Its incidence is increasing worldwide, particularly among the elderly and immunosuppressed [1, 2]. cSCCs typically occur in sun-exposed areas (e.g., the face, hands, and neck), but can appear anywhere. They encompass a spectrum ranging from in situ lesions to invasive forms that have the potential to destroy local tissue, invade perineurally, and, in high-risk cases, recur and metastasize, resulting in death [1, 2]. In situ cSCC is characterized by full-thickness epidermal dysplasia without penetration of the basement membrane and may progress to invasive cSCC if left untreated. Invasive SCC, on the other hand, is defined by dermal infiltration of atypical keratinocytes and can display features such as keratin pearl formation, variable degrees of differentiation, and an inflammatory stroma. Both types of cSCC exhibit a high mutational burden, which is predominantly driven by UV-induced DNA damage. Common genetic alterations include mutations in tumor suppressor genes such as *TP53, NOTCH1/2*, and *CDKN2A* [1, 2]. Additional molecular changes, such as epigenetic dysregulation, activation of tyrosine kinase pathways, alterations in genes involved in cell adhesion, epithelial-to-mesenchymal transition (EMT), and immunomodulation, are evidenced in the transition from in situ to invasive cSCC [1, 2]. Despite the variety of genetic and molecular changes observed, there are still no satisfactory molecular diagnostic biomarkers for high-risk cSCCs.

Most invasive cSCCs are treated with surgery, while advanced cases are treated with targeted or immunotherapy. However, the efficacy of epidermal growth factor receptor (EGFR) inhibitors is limited due to resistance and toxicity. Despite high response rates and durable remissions, the efficacy or applicability of anti-PD-1 is restricted in immunocompromised populations due to safety risks [1, 2]. Therefore, ongoing research is exploring combination strategies to improve cSCC therapies.

The tumor microenvironment (TME) plays a critical role in supporting cSCC progression, invasion, and immune evasion [3, 4]. Key components of the TME include cancer-associated fibroblasts (CAFs), immune cells, blood and lymphatic vessels, and the extracellular matrix (ECM). CAFs are prominent stromal cells that are reprogrammed by tumor-derived cytokines (e.g., TGF-β and IL-6), autocrine signaling, metabolic stress, and alterations in ECM stiffness and composition. In turn, CAFs contribute to tumor initiation, progression, and chemoresistance by secreting cytokines, growth factors, matrix metalloproteinases (MMPs), and ECM components. These secreted proteins facilitate epithelial cell proliferation, EMT, migration, invasion, and immune escape [5, 6].

Activated fibroblasts can promote EMT in tumor cells by secreting high levels of brain-derived neurotrophic factor (BDNF) [7, 8]. Both BDNF and its high-affinity receptor, neurotrophin receptor tyrosine kinase-2 (TrkB), are overexpressed in neurogenic and non-neurogenic tumors. High TrkB expression has been associated with cancer aggressiveness and poor prognosis [7]. Activation of TrkB can trigger multiple oncogenic signaling pathways, including PI3K/Akt, Ras/MAPK, JAK2/STAT3, and EMT-related transcription factors. This enhances proliferation, invasiveness, inflammation, and immune evasion [9–13]. STAT3 and IL-6 have emerged among these as pivotal downstream effectors. TrkB activates the JAK2/STAT3 pathway through multiple mechanisms, including stabilizing the JAK2 complex and inducing IL-6 to enhance JAK2 activity [14–18]. STAT3 can respond to and induce IL-6, creating a positive feedback loop that promotes tumor growth, inflammation, and metastasis [17, 19]. Although TrkB is frequently overexpressed in various tumors, little is known about its role in cSCCs.

Previously, we demonstrated that an age-related shift in the expression of BDNF and its receptor contributes to the increased susceptibility of aged skin to tumorigenesis [20]. Microenvironment-mediated TrkB activation in aged keratinocytes decreases E-cadherin and β-catenin expression, promotes the EMT program, and enhances proliferation and migration. Perturbation of the E-cadherin and/or catenin complex activates Yap1 and inhibits the Notch pathways, promoting proliferation, a stem-like phenotype, and invasiveness [20]. Our study revealed that TrkB activation plays a pivotal role in driving age-related keratinocyte plasticity and preneoplastic changes. This represents a critical step in the multistage progression toward cSCC and a potential pharmacologic target for preventing invasion or recurrence.

Based on our previous findings, this study aims to identify novel biomarkers and potential therapeutic targets for cSCC. We analyzed patient tumor samples to investigate the correlation between the expression of four specific proteins (i.e., TrkB, E-cadherin, Yap1, Notch1), clinicopathologic features, and cSCC risk. Additionally, we employed two human SCC cell lines and three primary CAF strains to evaluate the therapeutic efficacy of a TrkB-specific inhibitor in two-dimensional (2D) and three-dimensional (3D) models.

## Methods

### Patient selection

For immunohistochemical (IHC) analysis, we retrospectively selected patients diagnosed with either in situ or invasive cSCC at IDI-IRCCS. The inclusion criteria required that patients had a confirmed diagnosis of cSCC and a minimum of five years of clinical follow-up. Data on sex, age, and histological features were collected for each patient. The study population consisted of Italian patients of Caucasian ethnicity.

Patients were stratified into two groups based on lesion recurrence within the 5-year follow-up: low risk, for patients who developed only one SCC lesion; and high risk, for patients who developed multiple SCC lesions (≥ 4). All procedures were approved by the IDI-IRCCS Ethics Committee in Rome, Italy (protocol number 552/14-12-2018). Patient data were anonymized before analysis.

### Cell cultures and treatments

Human keratinocytes, fibroblasts, and CAFs were obtained from skin or SCC biopsies as previously described [21, 22]. Primary keratinocytes were cultivated on a feeder layer of lethally irradiated 3T3-J2 cells. 3T3-J2 cells (a gift from Prof Howard Green, Boston, MA), primary human keratinocytes, fibroblasts, and CAFs were grown as described previously [20, 21]. For further experiments, primary cells have been used at the second or third culture passage. SCC13 and 15 cells (a gift from James Rheinwald, Boston, MA) were grown on the feeder layer of 3T3-J2 as described previously [23].

For treatments, TrkB inhibitor ANA-12 (Cayman Chemical, Ann Arbor, MI) was used at the final concentrations of 50 μM and 100 μM on SCCs, and 50 μM on CAFs. SCCs, seeded onto the feeder layer, and CAFs were cultured for 48 h in complete medium. Cultures were treated for 7 days with medium replacement every 2 days. Culture treatments were performed in basal medium depleted of fetal calf serum supplemented with ANA-12. Subconfluent cultures were used for immunoblotting or quantitative RT-PCR analyses.

### Generation of skin equivalents & Invasion assay

For three-dimensional organotypic culture generation, 5 × 10^5^ fibroblasts or CAFs were embedded in 1 ml matrix gel [20]. After 1 h at 37 °C, gels were overlaid with 5 × 10^5^ primary keratinocytes or SCCs. Three-dimensional models were lifted at the air–liquid interface 24 h later, cultured for 7 days, and then fixed. Quantification of the invasion index was assessed by measuring the specific area of paraffin-embedded sections of the three-dimensional models stained with H&E using ImageJ software. The invasion index was calculated as 1 − (noninvading area/total area) [24]. These co-culture models were also treated with the ANA-12 (50 μM).

### Quantitative RT-PCR

RNA was extracted from cells by TRIzol (Invitrogen, Carlsbad, CA). Total RNA was reverse transcribed by an oligo(dT) primer (Aurogene, Rome, Italy). mRNA levels were analyzed by a QuantiTect SYBR Green-PCR kit (Qiagen, Hilden, Germany) by an ABI PRISM 7000 (Applied Biosystems, Foster City, CA). mRNA levels were normalized by the GUSB gene as the housekeeping gene. Primers are shown in Supplementary Table 1. Relative quantities of mRNA expression levels were calculated according to the 2 − ΔCt method or the 2 − ΔΔCt method.

### Immunoblot

The protein extracts from subconfluent SCCs or CAFs were prepared as previously described [25] and analyzed by SDS polyacrylamide gel electrophoresis using hand-made 7.5 and 12.5% gels or precast 4–20% gels (Bio-Rad, Laboratories, Inc., USA). Immunoblotting was performed as described previously [25]. Antibodies are indicated in Supplementary Table 2. Protein levels were evaluated by densitometric analysis and then normalized to GAPDH protein levels.

### Immunofluorescence and proliferation assay

SCCs and CAFs were seeded on round coverslips, cultured for 48 h in complete medium, and then treated with ANA-12.

For immunofluorescence, cells were fixed 24 h after treatments. Cells were fixed in 4% paraformaldehyde, and immunofluorescence was performed as previously described [26]. Antibodies are indicated in Supplementary Table 2. Nuclei were stained with DAPI. Images were captured using the ApoTome System connected to a Zeiss Axiovert 200 inverted microscope (Zeiss, Oberkochen, Germany), and the Nikon A1R + laser scanning confocal microscope (Nikon, Japan) with NIS Elements AR software.

For the proliferation assay, the 5-ethynyl-2'-deoxyuridine Alexa Fluor 488 Imaging Kit (Thermo Fisher, Waltham, MA) was used. Treated cultures were incubated with 5-ethynyl-2’-deoxyuridine (EdU) for 2–4 h. The cells were then fixed and counterstained with DAPI. All nuclei of 10 random fields (around 500 nuclei) per treatment were counted. The results are reported as EdU-positive nuclei/DAPI-stained nuclei.

### Immunohistochemistry and staining quantification

Immunohistochemistry (IHC) was carried out on formalin-fixed, paraffin-embedded (FFPE) tissue Sects. (4 μm) as described previously [25]. Antibodies are indicated in Supplementary Table 2.

The expression of TrkB and selected downstream targets (i.e., E-Cadherin, Yap1, and Notch1) was evaluated at both the epidermal and stromal levels (the latter only for invasive SCC) and quantified. Vimentin expression was evaluated as a control of stromal staining.

IHC results were evaluated by a semiquantitative approach used to assign an H-score to epidermal layers of each specimen of skin, SCC, or skin equivalent [27]. Briefly, staining intensity (0, 1 + , 2 + , or 3 +) was determined for each cell in a fixed field. Staining intensity was categorized as: 0 = negative, 1 +  = weak, 2 +  = moderate, 3 +  = strong). For each sample, at least 500 tumor or epidermal cells were evaluated in representative high-power fields (× 40 magnification). The H-score was assigned using the following formula: [1 × (% cells 1 +) + 2 × (% cells 2 +) + 3 × (% cells 3 +)], resulting in a score range of 0 to 300.

For stroma staining quantification, slides were scanned and imaged using the Mantra™ Quantitative Pathology Workstation (Akoya Biosciences). Image acquisition and spectral unmixing were performed using inForm® Advanced Image Analysis Software (Akoya Biosciences) to separate specific chromogenic signals from background. Regions of interest (ROIs) were selected across representative tumor areas. Quantification was performed by mean optical density within the stromal compartment. At least five non-overlapping fields per sample were analyzed to account for spatial heterogeneity, and at least 5000 tumor cells were evaluated.

### Migration assay

Migration was evaluated by OrisTM Cell Migration Kit (Platypus Technologies, Fitchburg, WI). Cells were seeded at high density (5 × 10^4^ per well) into 96-well plates containing silicon stoppers that restrict cells to an outer annular region and serum-starved overnight. The day after, stoppers were removed, and cells were treated. Each well was photographed by an EnSight Multimode Plate Reader (Perkin Elmer, UK) at different times. The cell-free area was measured by ImageJ software. The percentage of gap closure was calculated as 1‒ (cell-free area T/cell-free area T0) × 100.

### Secretome analysis

Supernatants were obtained from ANA-12 or untreated SCCs or CAFs at 70% of confluence, cultured in DMEM and 2% glutamine for 72 h. Supernatants were collected, clarified by centrifugation, and stored at − 80 °C for further analyses.

Collected supernatants were submitted to three biotin label‒based antibody arrays for profiling of protein secretion (RayBio C-Series Human Cytokine Antibody Array C6, RayBiotech, Norcross, GA). Protein levels were evaluated by densitometric analysis. Data analysis was performed as described previously [28].

### Dataset analysis

The raw data used for these analyses were obtained from the GEO dataset GSE144236 [29], accessed from https://www.ncbi.nlm.nih.gov/geo/query/acc.cgi?acc=GSE144236.

Data extraction and manipulation were performed in R (version 4.3.3), using the expression matrices and cell annotation files available from the GEO repository. Data were filtered and aggregated according to annotated cell clusters. Plots of the expression profiles for the four genes of interest were generated using ggplot2 and custom R functions.

### Statistical analyses

Demographic, clinicopathologic characteristics of patients with SCC and marker expression levels were described as means and standard deviations (SD), medians and Interquartile Ranges (IQR) for continuous variables (e.g., age, marker expression levels), and as absolute and relative frequencies for categorical variables (e.g., sex/gender, age groups, clinicopathological characteristics).

To compare differences between groups (e.g., SCC in situ vs. invasive), the Mann–Whitney U test was used for continuous variables, whereas the chi-square test or Fisher’s exact test, as appropriate, were used for categorical variables. Differences across “Grade groups” were assessed using Cuzick’s test for trend across ordered categories. Statistical analyses were performed using Stata software version 15 (StataCorp LLC, College Station, TX, USA) and OriginPro, Version 2020 (OriginLab Corporation, Northampton, MA, USA).

To evaluate the diagnostic ability of potential biomarkers to discriminate between two groups (i.e., in situ vs. invasive cSCCs, cSCC from low-risk vs. high-risk patients) and estimate their optimal cut-off values, Receiver Operating Characteristic (ROC) analysis was performed using GraphPad Prism (version 9.00). An Area Under the Curve (AUC), which measures overall accuracy, is considered indicative of good discriminatory ability of biomarkers if the value is above 0.7.

Experimental data on cells and 3D models were subjected to statistical analysis using appropriate tests and software (GraphPad Prism, version 9.00).

### Supervised machine learning algorithms

Several supervised machine learning algorithms were applied to the small datasets comprising selected protein expression extracted from two groups of tumor samples (i.e., in situ vs. invasive cSCCs, cSCC from low-risk vs. high-risk patients).

We tested four widely used classification algorithms:

1. *Logistic Regression (LR):* A supervised algorithm for categorical targets that estimates class membership probabilities using the logistic (sigmoid) function applied to a linear combination of input features. LR is efficient and performs well when there is a linear relationship between features and outcomes [30].
2. *Random Forest (RF):* An ensemble method that constructs multiple decision trees from random subsets of data and features. Predictions are aggregated by majority voting (classification) or averaging (regression), improving accuracy and reducing overfitting. RF captures complex, non-linear relationships through its randomized sampling [30].
3. *Support Vector Machine (SVM) with RBF Kernel:* SVM identifies the optimal hyperplane maximizing the margin between classes. The RBF kernel projects data into a higher-dimensional space to handle non-linear separability. This approach performed competitively, particularly in low-dimensional settings, though it requires careful hyperparameter tuning [31].
4. *K-Nearest Neighbors (KNN):* A simple algorithm that classifies samples based on the majority label among nearest neighbors. It performs well with clustered data but can struggle in high-dimensional spaces. Principal Component Analysis (PCA) was applied for exploratory visualization to identify patterns and outliers but was not used for dimensionality reduction in final models. An embedding-based KNN variant using sentence transformers was also tested, but showed slightly lower performance [30, 32].

## Results

### Clinicopathological features of patients with cSCC

These features are reported in Table 1. The majority of patients were male (69.1%). The average age was 80.2 years. Of the subjects, 56.4% were ≤ 80 years old and 43.6% were > 80 years old. The head was the most common anatomical site for cSCC, accounting for 38 out of 55 cases (69.1%). Specifically, the tumors were located on the face (36.4%), ear (20%), and scalp (12.7%). The other sites were the trunk (7.3%), the upper limb (10.9%), and the lower limb (12.7%). Tumors were classified as photoexposed (face, scalp, ears, dorsal forearms) (81,8%) or non-photoexposed (trunk, legs, inner arms) (18,1%) based on anatomical location and likelihood of chronic sun exposure. Finally, 65.4% of patients displayed multiple cSCCs.

**Table 1:** Clinicopathologic features of patients with SCC

Characteristics	All SCC		SCC in situ		SCC invasive		*P* value^a^
	(N = 55)		(N = 28)		(N = 27)		
	N	%	N	%	N	%	
**Sex**							
Males	38	69.1	18	64.3	20	74.1	
Females	17	30.9	10	35.7	7	25.9	0.562
**Age. y**							
Mean (SD)	80.2 (7.6)		79.4 (8.6)		81.0 (6.4)		
Median (IQR)	80 (75–86)		80 (74–85)		81 (75–85)		0.437^b^
≤ 80	31	56.4	18	64.3	13	48.1	
> 80	24	43.6	10	35.7	14	51.9	0.282
**Multiple SCC**	36	65.5	19	67.9	17	63.0	0.781
**Anatomic site**							
Head	38	69.1	20	71.4	18	66.7	
* Face*	*20*	*36.4*	*10*	*50.0*	*10*	*55.6*	
* Ear*	*11*	*20.0*	*4*	*20.0*	*7*	*38.9*	
* Scalp*	*7*	*12.7*	*6*	*30.0*	*1*	*5.6*	
Trunk	4	7.3	3	10.7	1	3.7	
Upper limb	6	10.9	2	7.1	4	14.8	
Lower limb	7	12.7	3	10.7	4	14.8	0.640
**Photoexposition**							
Photoexposed site	45	81,8	22	78,5	23	85,1	0.54
Non-photoexposed site	10	18,1	6	21,4	4	14,8	
**Grade**^*c*^							
G1			–		8	29.6	
G2			–		12	44.4	
G3			–		7	25.9	
**Stage**^*c*^							
pT1			–		19	76	
pT2			–-		6	24	
**Tumor thickness, mm**^**c**^							
≤ 2			–		15	60	
> 2					10	40	

SCC, Squamous Cell Carcinoma; SD, Standard Deviation; IQR, Interquartile Range

a: Fisher's exact test

b: Mann–Whitney U test

c: only for invasive SCC

The tumors were divided into two groups based on cSCC type: in situ cSCCs (28 out of 55 cases, or 50.9%) and invasive cSCCs (27 out of 55 cases, or 49.1%). According to histopathological assessment, in situ SCC corresponded to lesions confined to the epidermis, whereas invasive SCC was defined by the presence of dermal infiltration [33]. There were no significant differences between the two groups in terms of gender, age, anatomical site, photoexposition, or number of tumors. Based on histologic grade, 8 out of 27 invasive cSCCs were classified as G1 (29.6%), 12 as G2 (44.4%), and seven as G3 (25.9%). Pathological staging was performed according to TNM (UICC 2017). Among the invasive cSCCs, 76% of tumors were classified as pT1 and 24% as pT2; nodal (pN) and distant metastasis (pM) data were not available. Tumor thickness, recorded as the maximum vertical extent of the invasive component, was ≤ 2 mm in 60% of invasive cSCCs, whereas 40% of tumors measured > 2 mm.

Understanding the molecular differences between in situ and invasive cSCCs is essential for optimizing diagnostic accuracy and risk stratification.

### Expression and localization of TrkB, Yap1, E-Cadherin, and Notch1 in cSCCs

TrkB, encoded by the *NTRK2* gene, is overexpressed in some tumors, including oral and head and neck SCCs [7, 11, 34]. We previously demonstrated that TrkB activation in aged keratinocytes downregulates E-cadherin/β-catenin, triggering EMT and activating Yap1 while suppressing Notch, enhancing proliferation, stemness, and invasion [20]. Downregulation of the transmembrane glycoprotein E-cadherin is a typical trait of loss of cell polarity and cell-to-cell adhesion, as well as EMT [35]. Yap1 is activated by disrupted cell polarity, translocates into the nucleus, and promotes cell proliferation and plasticity [36]. In squamous epithelia, Notch1 generally functions as a tumor suppressor by promoting keratinocyte differentiation. Loss-of-function mutations in *NOTCH1* are among the most common genetic alterations observed in cSCCs [37].

**Fig. 1:**
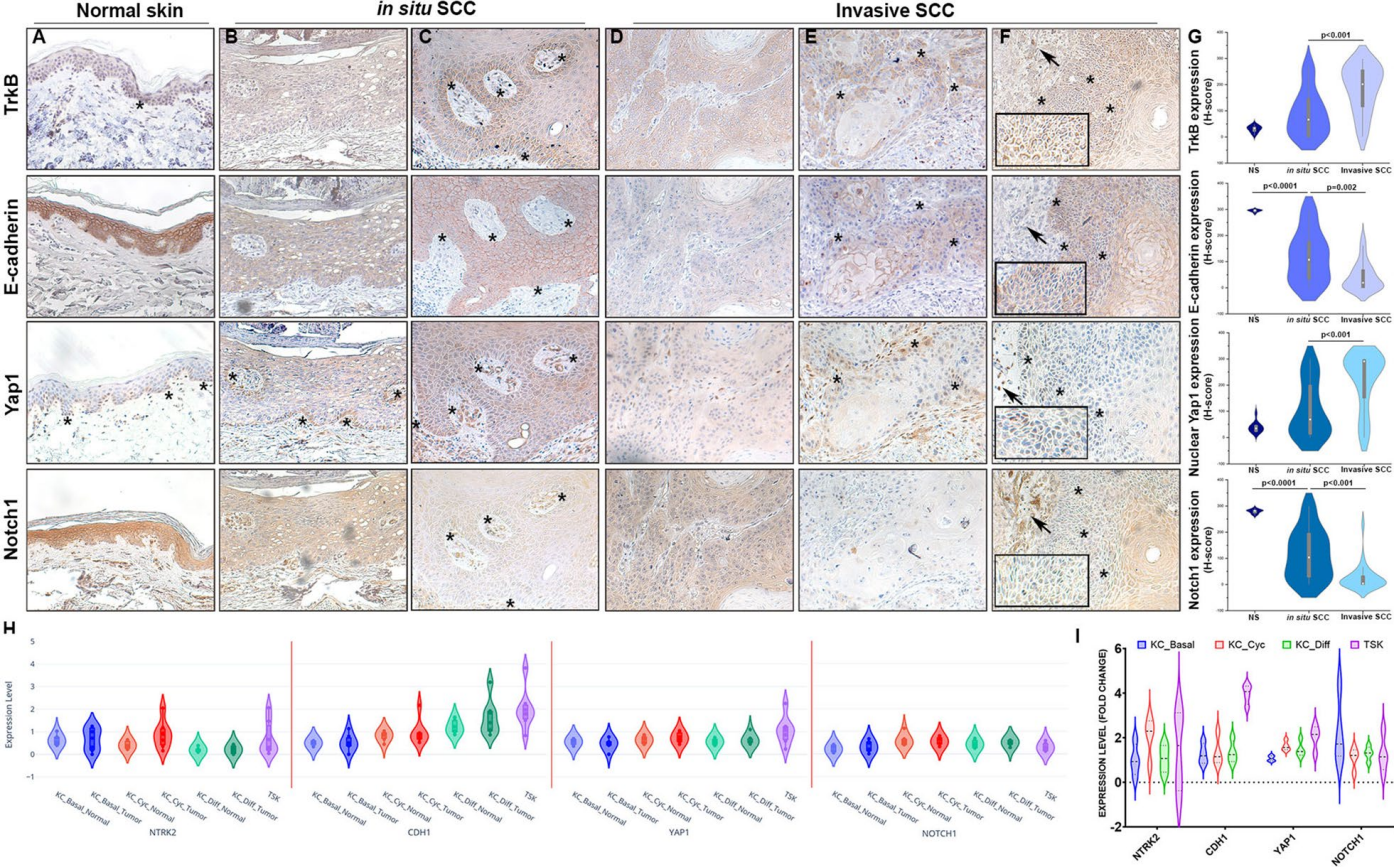
Expression and localization of TrkB, Yap1, E-Cadherin, and Notch1 in cSCCs. Representative images of paraffin-embedded specimens of normal skin (**A**), in situ (**B**, **C**), or invasive cSCC (**D**–**F**) immunostained with antibodies against TrkB, E-cadherin, Yap1, and Notch1 (20 × magnification; 40 × magnification for inset). The histological index (H-index) of each specimen was calculated (**G**). Data are shown as mean ± SD. Significance is determined using the Mann–Whitney U test and is indicated in the figure. **H** *NTRK2* (encoding TrkB), *CDH1* (E-cadherin), *YAP1* (Yap1), and *NOTCH1* (Notch1) transcript expression in normal skin and cSCC subpopulations from GSE144236 data sets. Three cSCC subpopulations recapitulate normal epidermal states (KC_Basal, KC_Cycling, and KC_Differentiated) and one is a tumor-specific keratinocyte (TSK) population. **I** Differential expression of tumor versus normal keratinocytes in patients who expressed *NTRK2*

We examined the expression of TrkB, E-cadherin, Yap1, and Notch1 using IHC. In normal, aged-matched skin (Fig. 1, column A), TrkB and Yap1 were restricted to a few basal cells (indicated by asterisks). Conversely, E-cadherin and Notch1 were highly expressed in differentiated keratinocytes, with E-cadherin localized to the membrane. SCCs displayed heterogeneous protein expression patterns. Some in situ cSCCs did not express TrkB (25.8%) (Fig. 1, column B). In these specimens, E-cadherin expression was lower compared to normal skin, but it was still localized to the membrane. Yap1 was expressed in a few cells at the edge of the tumor (asterisks), whereas Notch1 was expressed in the lesion, albeit at lower levels than in skin. Most in situ cSCCs (71.4%) expressed TrkB; its localization was often at the tumor border (Fig. 1, column C, asterisks). In these marginal areas, E-cadherin and Notch1 were absent, while nuclear Yap1 was highly expressed.

All invasive cSCCs exhibited TrkB expression. A small subset of tumors (14.8%) displayed membrane-associated TrkB expression (Fig. 1, column D). In these specimens, E-cadherin and Yap1 were not expressed, while Notch1 was present. Conversely, in most cSCCs, TrkB was expressed at the edge of the tumor (Fig. 1, column E, asterisks), where the cells displayed EMT-like morphology. In these cells, TrkB was localized both in the membrane and the cytoplasm, as has been seen in other tumors [7, 11, 34]. We found that E-cadherin was absent from cell–cell junctions and exhibited only a low cytoplasmic signal, Notch1 was reduced, whereas Yap1 was expressed in the nuclei of TrkB-positive tumor marginal areas. Similar nuclear Yap1 staining at the invasive edge was observed in SCCs [36, 38]. Notably, some specimens exhibited a multilayered edge, in which this pattern was more evident (Fig. 1, column F, asterisks and insets). In line with these results, a study found significantly increased mRNA expression of TrkB and EMT markers, alongside decreased expression of E-cadherin, encoded by the *CDH1* gene, at the tumor edge of SCCs [39].

To complement our findings, we analyzed raw data from the Gene Expression Omnibus (GEO) dataset GSE144236 [29]. This allowed us to cross-validate the four analyzed markers and gain additional insight into their transcription and localization. To characterize cSCCs, the authors integrated single-cell RNA sequencing, spatial transcriptomics, and multiplexed imaging [29]. They identified four tumor subpopulations: three that recapitulated normal epidermal states (KC_Basal, KC_Cycling, and KC_Differentiated) and one tumor-specific keratinocyte (TSK) population. The TSK population was characterized by an EMT-like signature and was localized to the leading tumor edges. We selected data corresponding to the cell types of interest and focused on the expression levels of the *NTRK2*, *CDH1*, *YAP1*, and *NOTCH1* genes across the identified cellular clusters. We found that TSKs exhibit increased transcripts of *NTRK2*, *CDH1*, and *YAP1* (Fig. 1H). Next, we analyzed the differential expression of tumor versus normal keratinocytes in patients who expressed the *NTRK2* transcript. TSKs were compared to normal basal cells (Fig. 1I). As evidenced by protein expression at the tumor edge (Fig. 1, column F, insets), *NTRK2* transcription was predominantly upregulated in cycling tumor keratinocytes and TSKs. High *CDH1* transcripts were present in TSKs. An increase or stability in E-cadherin mRNA accompanied by a decrease in protein levels is common in tumors, due to post-transcriptional, translational, or post-translational mechanisms. This uncoupling is often observed during the EMT process when E-cadherin protein is lost at cell-to-cell contacts [35]. *YAP1* transcripts were upregulated, whereas *NOTCH1* was downregulated in cycling tumor cells and TSKs. This pattern is similar to what we observed at the invasive edge.

Thus, EMT-like cells at the invasive cSCC edge exhibit high TrkB and nuclear Yap1 protein expression alongside decreased E-cadherin and Notch1 levels.

### Correlation between protein expression levels and clinicopathological features in patients with cSCC

TrkB, E-cadherin, Yap1, and Notch1 expression levels were quantified using the H-score method (Table 2). Supplementary Fig. 1 shows that TrkB expression significantly correlates with decreased E-cadherin expression (r = − 0.291, *p* = 0.031) and increased nuclear Yap1 expression (r = 0.501, *p* < 0.001). Decreased E-cadherin expression significantly correlated with increased nuclear Yap1 levels (r = − 0.340, *p* = 0.011) and decreased Notch1 levels (r = 0.549, *p* < 0.001), which reinforces the hypothesis that TrkB activation can drive cSCC tumorigenesis through the Yap1 and Notch1 pathways. A significant inverse correlation was also observed between nuclear Yap1 and Notch1 expression (r = − 0.480; *p* < 0.001).

**Table 2:** Expression of TrkB, Yap1, E-Cadherin, Notch1 and Vimentin in in situ vs invasive SCCs

Epidermal layers	All SCC	SCC in situ	SCC invasive	*P* value^a^
	(N = 55)	(N = 28)	(N = 27)	
	N %	N %	N %	
**TrkB**				
mean (SD)	137.2 (99.6)	90.3 (87.0)	185.8 (89.0)	
median (IQR)	133.3 (50.0–236.4)	66.3 (0.0–147.6)	201.9 (115.0–257.0)	< 0.001
**Yap1nuclear**				
mean (SD)	161.8 (120.2)	106.2 (105.2)	219.5 (108.3)	
median (IQR)	180.0 (32.5–294.0)	68.7 (12.5–200.4)	291.0 (150.2–298.0)	< 0.001
**E-cadherin**				
mean (SD)	82.8 (87.2)	120.6 (96.8)	43.5 (53.9)	
median (IQR)	58.3 (4.0–150.0)	107.3 (32.7–178.0)	19.3 (0.0–69.6)	0.002
**Notch1**				
mean (SD)	71.4 (87.1)	115.4 (94.3)	25.8 (47.9)	
median (IQR)	33.3 (0.0–126.2)	103.5 (27.6–196.0)	4.0 (0.0–33.3)	< 0.001

SCC, Squamous Cell Carcinoma; SD, Standard Deviation; IQR, Interquartile Range

a: Mann–Whitney U test

b: only for invasive SCC

The potential correlation between TrkB, E-cadherin, Yap1, and Notch1 levels and clinicopathological features was then investigated. No significant associations were found between protein expression and characteristics such as gender, age, anatomical site, grade, stage, or tumor thickness (Supplementary Tables 3 and 4). However, an association was found between the anatomic site and nuclear Yap1 expression (*p* = 0.027). Tumors from the head exhibited higher nuclear Yap1 expression. Different staining patterns were observed among the photoexposed and non-photoexposed sites; however, these differences did not reach statistical significance, likely due to the unequal group sizes (45 vs 10 samples) (Supplementary Fig. 2A).

Considering the different cSCC types (Fig. 1, column G, and Table 2), we found that TrkB and nuclear Yap1 expression levels were significantly higher in invasive cSCCs than in situ cSCCs (*p* < 0.001). Conversely, E-cadherin and Notch1 expression were significantly lower in invasive cSCCs than in situ cSCCs (*p* = 0.002 and *p* < 0.001, respectively). Their expression was also lower in in situ cSCCs than in normal skin (*p* < 0.0001).

Thus, TrkB, E-cadherin, Yap1, and Notch1 protein expression levels mainly correlate with cSCC types.

### Epithelial signature for discrimination of in situ cSCC vs invasive cSCC

**Fig. 2:**
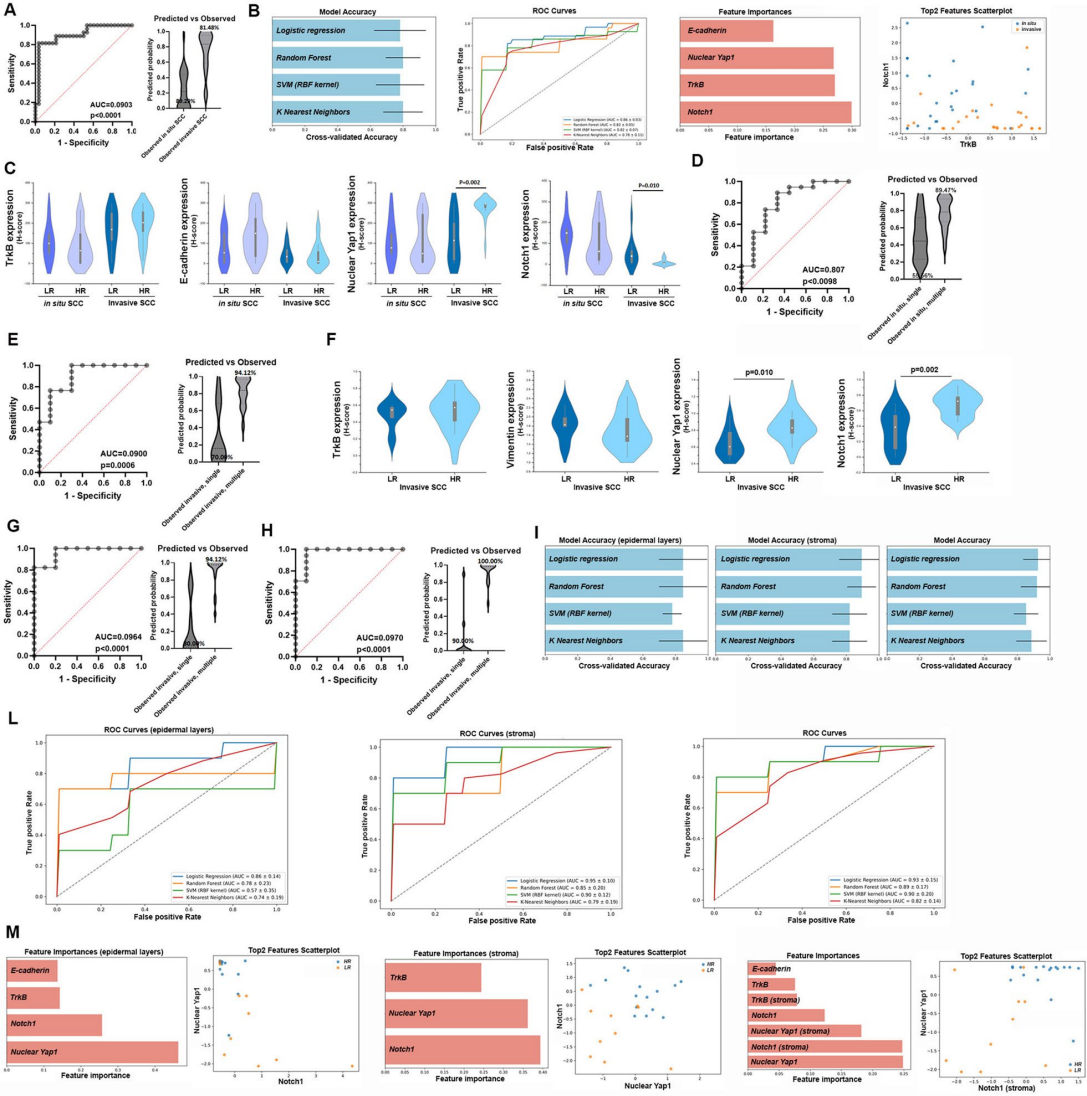
*‘TrkB, E-cadherin, nuclear Yap1, Notch1’* signature for discrimination of cSCC type and invasive cSCC risk. **A** ROC curve on combined expression values of TrkB, E-cadherin, Yap1, and Notch1 in in situ vs invasive cSCCs. **B** Model comparison of supervised machine learning algorithms applied to expression values of TrkB, E-cadherin, Yap1, and Notch1 in in situ vs invasive cSCCs. Model accuracy, ROC curves, Feature importance, and Top2 feature scatter plot are reported. **C** Differential expression of TrkB, E-cadherin, Yap1, and Notch1 in low-risk (LR) and high-risk (HR) in situ or invasive cSCCs. Data are shown as mean ± SD. Significance was determined using the Mann–Whitney U test. Significance is indicated in the figure. **D**–**E** ROC curve on combined expression values of TrkB, E-cadherin, Yap1, and Notch1 in low-risk (single) vs high-risk (multiple) in situ or invasive cSCCs. **F** Differential expression of stromal TrkB, E-cadherin, Yap1, and Notch1 in low-risk (LR) and high-risk (HR) invasive cSCCs. Data are shown as mean ± SD. Significance was determined using the Mann–Whitney U test. Significance is indicated in the figure. **G**–**H** ROC curve on combined expression values of TrkB, E-cadherin, Yap1, and Notch1 datasets (i.e., stroma values, epidermal layers + stroma values) in low-risk (single) vs high-risk (multiple) invasive cSCCs. Model comparison of supervised machine learning algorithms applied to our datasets (i.e., epidermal layer values, stroma values, epidermal layers + stroma values) of low-risk vs high-risk invasive cSCCs. Model accuracy (**I**), ROC curves (**L**), Feature importance, and Top2 feature scatter plot (**M**) are reported

ROC analysis revealed that TrkB, E-cadherin, Yap1, and Notch1 are effective biomarkers for discriminating between in situ and invasive cSCCs, with respective AUC values of 0.797 (*p* < 0.001), 0.739 (*p* = 0.002), 0.765 (*p* = 0.001), and 0.801 (*p* < 0.001) (Supplementary Fig. 2B). Multiple logistic regression analysis revealed that the *‘TrkB, E-cadherin, nuclear Yap1, Notch1’* signature displayed high diagnostic power (AUC = 0.903, *p* < 0.0001) for discriminating cSCC types. Notably, 89.29% of observed in situ cSCCs and 81.48% of observed invasive SCCs were correctly classified (Fig. 2A).

To validate this finding, several supervised machine learning algorithms were applied to our small dataset to explore the discriminative power of the selected biomarkers, compare the generalization performance of standard classifiers, and establish whether consistent and interpretable predictive signatures could be identified. Small datasets are vulnerable to overfitting, and reported accuracies, especially when models are evaluated using the same data on which they were trained, can be overly optimistic. For this reason, all models were evaluated using k-fold cross-validation, and care was taken not to overinterpret the results.

We tested four widely used classification algorithms: Logistic Regression (LR), Random Forest (RF), Support Vector Machine (SVM) with RBF Kernel, K-Nearest Neighbors (KNN)(see Methods section)[30–32]. Multiple classifiers were evaluated using cross-validation (CV) to assess not just raw accuracy, but also model stability and variance. This comparative analysis provides a framework for selecting models suited to similar datasets in the future. All four classifiers achieved competitive performance, with CV accuracies ranging from ~ 78% to over 90% (Fig. 2B, blue columns). RF was consistently competitive and offered the advantage of interpretability via feature importance analysis, even if LR or KNN achieved higher accuracy in some tasks (Fig. 2B, blue columns). RF slightly outperformed the others in stability and interpretability. ROC curve analysis yielded AUC values between approximately 0.78 and 0.86 across tasks, reflecting good discriminatory capacity of the trained models (Fig. 2B, ROC curve).

By analyzing model outputs such as feature importance, we can identify which biomarkers contributed most significantly to the classification. This can then inform further experiments or clinical focus. Feature importance analysis identified Notch1, Yap1 nuclear, and TrkB as the top contributors (Fig. 2B, orange, columns). E-cadherin showed a lower influence in this classification task. PCA projections of the dataset onto the first two top contributors (Notch1 and TrkB) provided additional qualitative support for the discriminative capacity of the selected biomarkers and validated the choice of features used in the classification models (Fig. 2B, PCA).

Thus, the specific signature *‘TrkB, E-cadherin, nuclear Yap1, Notch1’* can be an interesting biomarker for discriminating cSCC types.

### Epithelial signature for discrimination of cSCC risk

The use of IHC-based signatures to evaluate SCC risk has become an important tool in research and clinical settings. The median time of cSCC recurrence is consistently between 9 and 13 months, with the vast majority (≥ 95%) occurring within two to three years. During this time, actinic keratoses or in situ cSCCs can progress to invasive forms [40–43].

Specimens were collected from patients with different oncologic histories: n = 9 developed a single in situ cSCC; n = 19 developed ≥ 4 in situ or invasive cSCCs; n = 10 developed a single invasive cSCC; and n = 17 developed ≥ 4 invasive cSCCs. The follow-up period was 5 years. Thus, specimens were stratified based on patient SCC risk. Considering in situ cSCCs, TrkB, nuclear Yap1, and Notch1 expression were reduced in lesions from high-risk patients, coinciding with an increase in E-cadherin (Fig. 2C and Table 3). Conversely, TrkB and nuclear Yap1 levels were higher (*p* = 0.002) in invasive cSCCs from high-risk patients, whereas E-cadherin and Notch1 amounts were lower (*p* = 0.010).

**Table 3:** Association between the expression levels of Trkb, E-cadherin, Yap1, Notch1, Vimentin and SCC risk

	SCC in situ			SCC invasive		
***Epidermal layers***:	Single (N = 9)	Multiple (N = 19)	*P* value^a^	Single (N = 10)	Multiple (N = 17)	*P* value^a^
**TrkB**						
Mean (SD)	101.0 (86.9)	85.3 (88.9)		167.1 (100.1)	196.8 (83.0)	
Median (IQR)	100.0 (50.0–147.2)	64.0 (0.0–148.0)	0.550	168.5 (115.0–250.1)	202.9 (158.5–257.0)	0.393
**Yap1nuclear**						
Mean (SD)	114.5 (100.2)	102.2 (109.9)		126.9 (116.9)	274.0 (53.6)	
Median (IQR)	78.0 (49.8–150.0)	49.8 (5.2–244.7)	0.622	114.4 (17.9–200.6)	296.4 (276.0–298.0)	0.002
**Ecadherin**						
Mean (SD)	79.9 (83.5)	139.9 (98.7)		44.0 (46.2)	43.2 (59.4)	
Median (IQR)	54.0 (0.0–150.0)	150.0 (34.0–225.0)	0.109	34.4 (6.0–69.6)	10.0 (0.0–60.0)	0.574
**Notch1**						
Mean (SD)	138.7 (80.6)	104.3 (100.2)		55.2 (69.1)	8.6 (13.9)	
Median (IQR)	150.0 (100.0–168.3)	62.1 (19.7–200.0)	0.388	39.8 (7.7–67.0)	2.8 (0.0–9.0)	0.010
***Stroma***^***b***^						
**TrkB**						
Mean (SD)				0.5 (0.2)	0.5 (0.2)	
Median (IQR)				0.5 (0.5–0.6)	0.6 (0.4–0.6)	0.451
**Yap1nuclear**						
Mean (SD)				0.6 (0.2)	0.8 (0.2)	
Median (IQR)				0.6 (0.5–0.8)	0.8 (0.8–0.9)	0.010
**Vimentin**						
Mean (SD)				1.8 (0.3)	1.7 (0.4)	
Median (IQR)				1.8 (1.8–2.0)	1.6 (1.5–2.0)	0.346
**Notch1**						
Mean (SD)				0.3 (0.2)	0.7 (0.2)	
Median (IQR)				0.4 (0.1–0.5)	0.7 (0.5–0.8)	0.002

SCC, Squamous Cell Carcinoma; SD, Standard Deviation; IQR, Interquartile Range

a: Mann–Whitney U test

b: only for invasive SCC

To identify the ability of these biomarkers to discriminate cSCC risk, ROC analyses were performed. Regarding in situ cSCCs, no proteins can be considered good biomarkers, as they exhibited an AUC < 0.7 (Supplementary Fig. 2C). Multiple logistic regression analysis using a combination of these four proteins yielded an AUC value of 0.807 (*p* = 0.0098). While 89.47% of observed in situ cSCCs from high-risk patients were correctly classified, only 55.56% of observed lesions from low-risk patients were properly categorized (Fig. 2D). Therefore, the *‘TrkB, E-cadherin, nuclear Yap1, Notch1’* signature did not exhibit good diagnostic power to discriminate tumor risk in patients with in situ cSCCs.

For invasive cSCCs, the results indicated that Notch1 is the only effective biomarker for discriminating between low- and high-risk patients (AUC = 0.794; *p* = 0.012; Supplementary Fig. 2D). However, multiple logistic regression analysis combining the four proteins indicated that the *‘TrkB, E-cadherin, nuclear Yap1, Notch1’* signature displayed high diagnostic power (AUC = 0.900, *p* = 0.0006) for discriminating invasive cSCC risk. Notably, 70% of the observed invasive cSCCs from low-risk patients and 94.12% of the observed lesions from high-risk patients were correctly classified (Fig. 2E).

Thus, the epithelial signature *‘TrkB, E-cadherin, nuclear Yap1, Notch1’* could be an interesting biomarker for discriminating invasive cSCC risk.

### Stromal signatures for determination of invasive cSCC risk.

Notch signaling exhibits highly context-dependent functions that vary across cell types, developmental stages, and normal versus pathological conditions. Both Yap1 and Notch1 signaling pathways are activated in most stromal cells, including CAFs and immune cells, and regulate the intricate communication between tumor and stromal cells in multiple ways [36, 44].

We quantified the expression of TrkB, Yap1, Notch1, and vimentin (as an internal control) in the TME of invasive cSCCs. Their expression was higher compared to normal skin (Supplementary Fig. 2E and Fig. 1, column F, arrows). We did not observe a difference in TrkB expression in invasive cSCCs from low- and high-risk patients (Fig. 2F). A significant increase (*p* = 0.005) was found only in specimens from females (Supplementary Table 3). Notably, a significant increase in nuclear Yap1 (*p* = 0.010) and Notch1 (*p* = 0.002) was observed in specimens from high-risk patients (Fig. 2F). Interestingly, vimentin expression decreased slightly in high-risk patients, and its levels significantly correlated with tumor grade (*p* = 0.013; Supplementary Table 3). No significant associations were found between protein expression in the stroma and photoexposition (Supplementary Fig. 2A), tumor stage or tumor thickness (Supplementary Table 4).

To evaluate the diagnostic value of stromal biomarkers (i.e., TrkB, Yap1, and Notch1) in predicting the risk of invasive cSCCs, a ROC analysis was conducted. The results indicated that Yap1 and Notch1 are good stromal biomarkers for discriminating between low- and high-risk patients with invasive cSCCs (AUC = 0.794, *p* = 0.012 and AUC = 0.864, *p* = 0.069, respectively; Supplementary Fig. 2F). Multiple logistic regression analysis using a combination of these proteins indicated that the stromal signature *‘TrkB, nuclear Yap1, Notch1’* displayed high diagnostic power (AUC = 0.964, *p* < 0.0001) for discriminating invasive cSCC risk. Notably, 80% of the observed invasive cSCCs from low-risk patients and 94.12% of the observed lesions from high-risk patients were correctly classified (Fig. 2G).

Thus, the specific stromal signature *‘TrkB, nuclear Yap1, Notch1’* could be an interesting biomarker for discriminating invasive cSCC risk.

### Global signature for discrimination of invasive cSCC risk

To refine the identification of a strong diagnostic signature, data on the expression levels of biomarkers in tumor and stromal cells were used in a multiple logistic analysis. The results indicated that the global signature *‘TrkB, E-cadherin, nuclear Yap1, Notch1’* had the greatest diagnostic power (AUC = 0.970, *p* < 0.0001) for discriminating invasive SCC risk. Notably, 90% of the observed invasive cSCCs from low-risk patients and 100% of the observed lesions from high-risk patients were correctly classified (Fig. 2H).

To validate these findings, model comparison of supervised machine learning algorithms was applied to our small datasets (i.e., epidermal layer values, stroma values, epidermal layers + stroma values). The performance of evaluated classifiers varied across the three related tasks in classifying different subtypes (Fig. 2I). Considering epidermal layer values, CV accuracies ranged from 78 to 85%, with KNN and LR showing the highest performance. RF achieved a CV accuracy of ~ 81%, and a test accuracy of 0.82. Evaluating stromal data, models performed well. Both RF and LR reached a CV accuracy of ~ 89%, and test accuracy was 0.73. Including stromal data to epidermal layer values significantly boosted model performance, with LR reaching a CV accuracy of ~ 93%, followed by KNN (~ 89%) and SVM (~ 85%).

ROC analyses (Fig. 2L) generated AUC values ranging from approximately 0.75 to 0.92 across the different tasks, indicating a strong discriminatory ability of the trained models. Notably, the highest AUC was observed in the classification task incorporating epidermal and stromal features, reinforcing the enhanced predictive value of stromal markers.

Feature importance analysis from the optimized RF models consistently identified TrkB, nuclear Yap1, and Notch1 as the most influential markers across classification tasks (Fig. 2M). In contrast, E-cadherin demonstrated lower importance scores, suggesting a more nuanced or less direct role within the current datasets. The inclusion of stromal markers further refined the model, improving accuracy and emphasizing the predictive value of the tumor microenvironment. PCA analyses of the “top 2 features” of each task were reported. Notably, including the stroma dataset, feature importance shifted towards nuclear Yap1 (epidermal layers) and Notch1 (stroma) as dominant contributors.

Thus, the specific signature *‘TrkB, nuclear Yap1, Notch1’* can be a strong biomarker for invasive cSCC risk. Despite the limited sample size, machine learning models demonstrated the ability to differentiate between SCC classes with promising accuracy.

#### TrkB inhibition and EMT-like phenotype

To evaluate TrkB pathway activation at the tissue level, we assessed phosphorylated TrkB (p-TrkB) and its ligand BDNF on histological sections. As shown in Supplementary Fig. 3A, TrkB expression was predominantly observed in keratinocytes at the tumor rim and in mesenchymal stromal cells (black arrows). Among TrkB-positive tumors, p-TrkB expression was detected in keratinocytes in 73.7% of cases and in mesenchymal cells in 89.5%, indicating signaling activation in most tumors. BDNF expression was observed in some keratinocytes in 21.1% of cases and in mesenchymal cells in all samples (100%) (black arrows). Notably, strong BDNF expression was also detected in vascular endothelial cells (red arrows).

Given the observed activation of TrkB signaling in tumor keratinocytes and stromal cells, we next evaluated TrkB as a potential therapeutic target for SCC by examining the effects of its pharmacological inhibition in two SCC cell lines with distinct *TP53* alterations and different characteristics (Supplementary Table 5). SCC13 carries a point mutation (c.772 G → A) that affects the DNA-binding domain, while SCC15 harbors a splice-site insertion that disrupts normal transcript processing, resulting in the absence of the protein [45, 46]. Both alterations result in *TP53* loss-of-function, which matches the broader landscape of p53 inactivation typical of cSCC [1], where p53-mediated tumor suppression is compromised. SCC13 cells have higher colony-forming efficiency and proliferation rate than SCC15 [47]. Both SCC13 and SCC15 cells can undergo reversible and irreversible growth arrest; however, only SCC15 cells are capable of significant terminal differentiation. SCC13 cells are unable to complete the final stage of differentiation, specifically the formation of the cornified envelope [48].

**Fig. 3:**
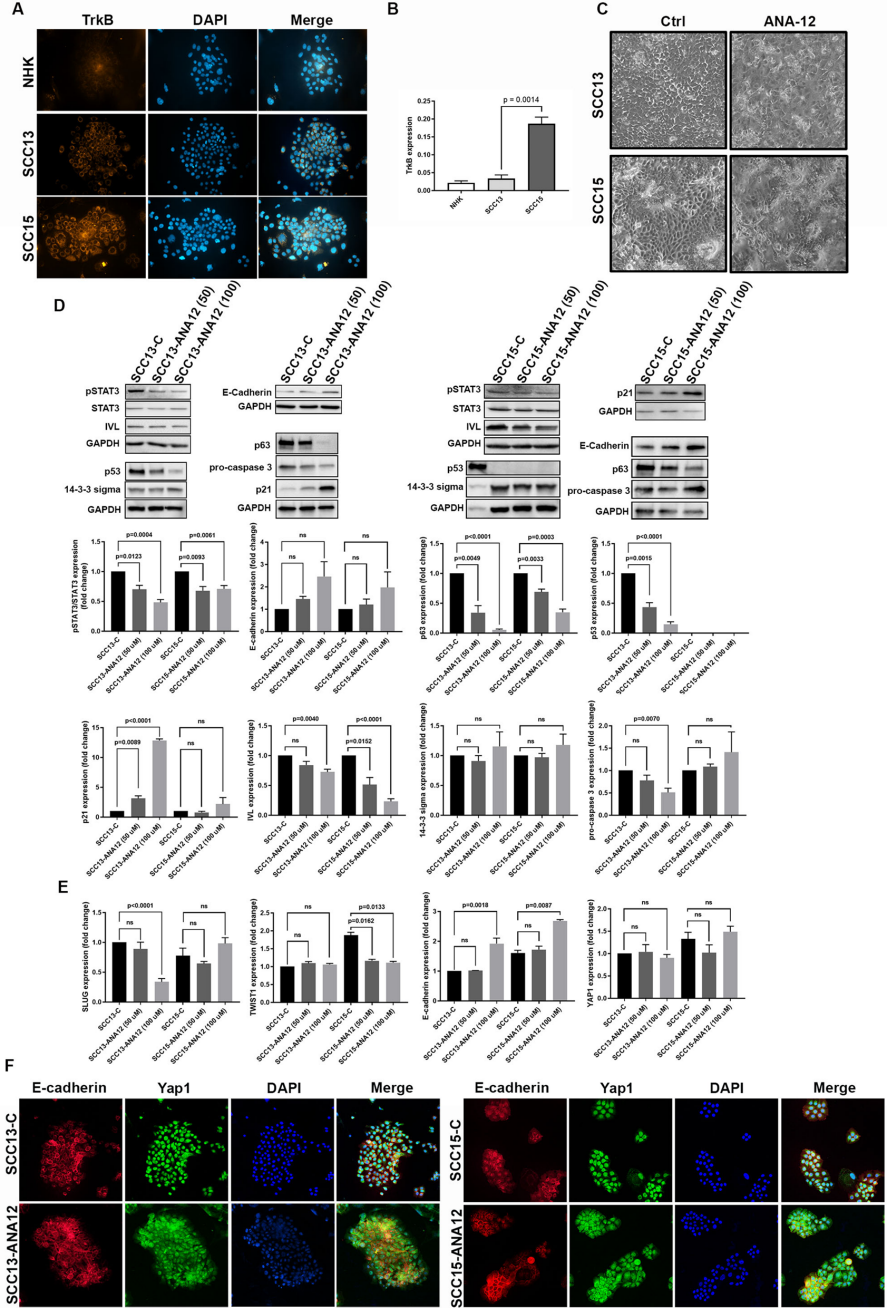
TrkB inhibition reduces the EMT-like phenotype of SCC cells. **A** Representative images of TrkB immunostaining (orange) in primary normal human keratinocytes (NHK) and SCC cells. DAPI (blue) was used for nuclear staining. **B** Relative TrkB expression in NHK and SCC cells by RTqPCR. **C** Representative images of untreated (Ctrl) or treated (ANA-12) SCC13 and SCC15 cells. **D** Representative immunoblots and related densitometric values (n = 3) of pSTAT3, STAT3, E-cadherin, involucrin (IVL), 14-3-3σ, p63, p53, p21 and pro-caspase 3 in untreated vs ANA-12-treated SCC. GAPDH was the loading control. A431 was used as a positive control for p53. **E** Fold-change expression of Slug, Twist1, E-cadherin, and Yap1 in untreated vs ANA-12-treated SCC cells by RTqPCR. **F** Representative images of E-cadherin (red) and Yap1 (green) immunostaining in untreated vs ANA-12-treated SCC cells. DAPI (blue) was used for nuclear staining. All data are shown as mean ± SD. Significance was determined using a two-tailed Student’s t-test and is indicated in the figure. ns = not significant. Images were acquired at 20 × magnification

As shown in Fig. 3A, both SCC lines expressed TrkB protein, which is mainly localized in the cytoplasm of EMT-like cells as observed in SCC histological specimens. After neurotrophin binding, the TrkB receptor is internalized by endocytosis, and, thus, a consistent amount of the protein is located in intracellular pools and released in an activity-dependent manner [49]. SCC15 exhibited a higher TrkB expression compared to SCC13 at both transcriptional and translational levels (Fig. 3A and B). P-TrkB was also detected, confirming that the receptor is in an activated state (Supplementary Fig. 3B). Moreover, BDNF expression was higher in SCC cells than in keratinocytes from age-matched healthy donors, with SCC13 showing the highest levels (Supplementary Fig. 3C), supporting the presence of TrkB ligand in culture.

TrkB activates several signaling pathways (i.e., Ras/MAPK, PI3K/AKT, and JAK2/STAT3) that converge to induce EMT transcription factors such as Snail, Slug, and Twist [17, 50, 51], leading to repression of E-cadherin [51]. Since E-cadherin–mediated junctions activate the Hippo pathway and inhibit Yap1 through phosphorylation and cytoplasmic retention, loss of E-cadherin results in increased nuclear Yap1 activity and transcriptional signaling [52].

SCCs exhibited an EMT-like morphology with wide cellular interspace, indicating reduced cell–cell adhesion properties (Fig. 3C). SCC13 cells were homogeneous in size and grew in a monolayer, whereas SCC15 cells were more heterogeneous in size, with both small proliferating and enlarged differentiating cells. SCC13 expressed lower levels of E-cadherin and differentiation markers, such as 14-3-3σ and involucrin, than SCC15 (Supplementary Fig. 4A). Notably, although SCC13 expresses lower levels of TrkB compared with SCC15, both cell lines exhibit EMT-like features, indicating that EMT is not solely dependent on TrkB expression levels but rather on its activation status. Differences in the tumor microenvironment, including anatomical site–specific characteristics and ligand availability, such as higher BDNF levels in SCC13, may contribute to differential activation of TrkB-dependent pathways by autocrine or paracrine signaling.

The SCCs were treated with a selective TrkB inhibitor (ANA-12), which we had previously characterized in primary skin cells [20]. Following treatment, both SCC cell lines exhibited a loss of EMT-like morphology and a significant reduction in cellular interspace (Fig. 3C). SCC15 cells formed a higher number of stratified colonies, resembling normal keratinocytes. ANA-12 treatments significantly reduced the phosphorylation of its target STAT3 in both SCC types (Fig. 3D). Although total ERK and AKT protein levels decreased, only a slight trend toward reduced pERK/ERK ratio was noted, and no significant change in the pAKT/AKT ratio was observed (Supplementary Fig. 4B). ANA-12 treatment significantly reduced Slug transcription in SCC13 and Twist1 transcription in SCC15 (Fig. 3E) and, in turn, induced E-cadherin expression at the transcriptional and translational levels (Fig. 3D and E). As shown in Fig. 3F E-cadherin was present in the cytoplasm of both SCC cell lines and was properly localized to the membrane after ANA-12 treatment. Concurrently, Yap1 translocated from the nucleus to the cytoplasm, presumably due to the restoration of cell-to-cell contact. Indeed, administration of ANA-12 did not significantly alter Yap1 transcription (Fig. 3E).

Thus, TrkB inhibition suppresses key oncogenic signaling pathways, reduces the EMT-like phenotype of SCC cells, restores cell–cell adhesion, and sequesters Yap1 in the cytoplasm, preventing the activation of pro-proliferative and cell plasticity programs.

#### TrkB inhibition and SCC proliferation and migration

EMT inhibition limits tumor growth by reversing stemness [51, 53]. ΔNp63, the major p63 isoform in epithelial tissues, promotes proliferation and stem cell maintenance while preventing senescence and differentiation [54–56]. STAT3 directly regulates ΔNp63 expression, and together they control cancer stem cell maintenance [57–59]. The ACTL6A/ΔNp63 complex enhances proliferation and inhibits differentiation by activating Yap signaling [60]. ΔNp63 also suppresses the cyclin-dependent kinase inhibitor (CDKI) p21, thereby promoting cell survival and proliferation [61].

**Fig. 4:**
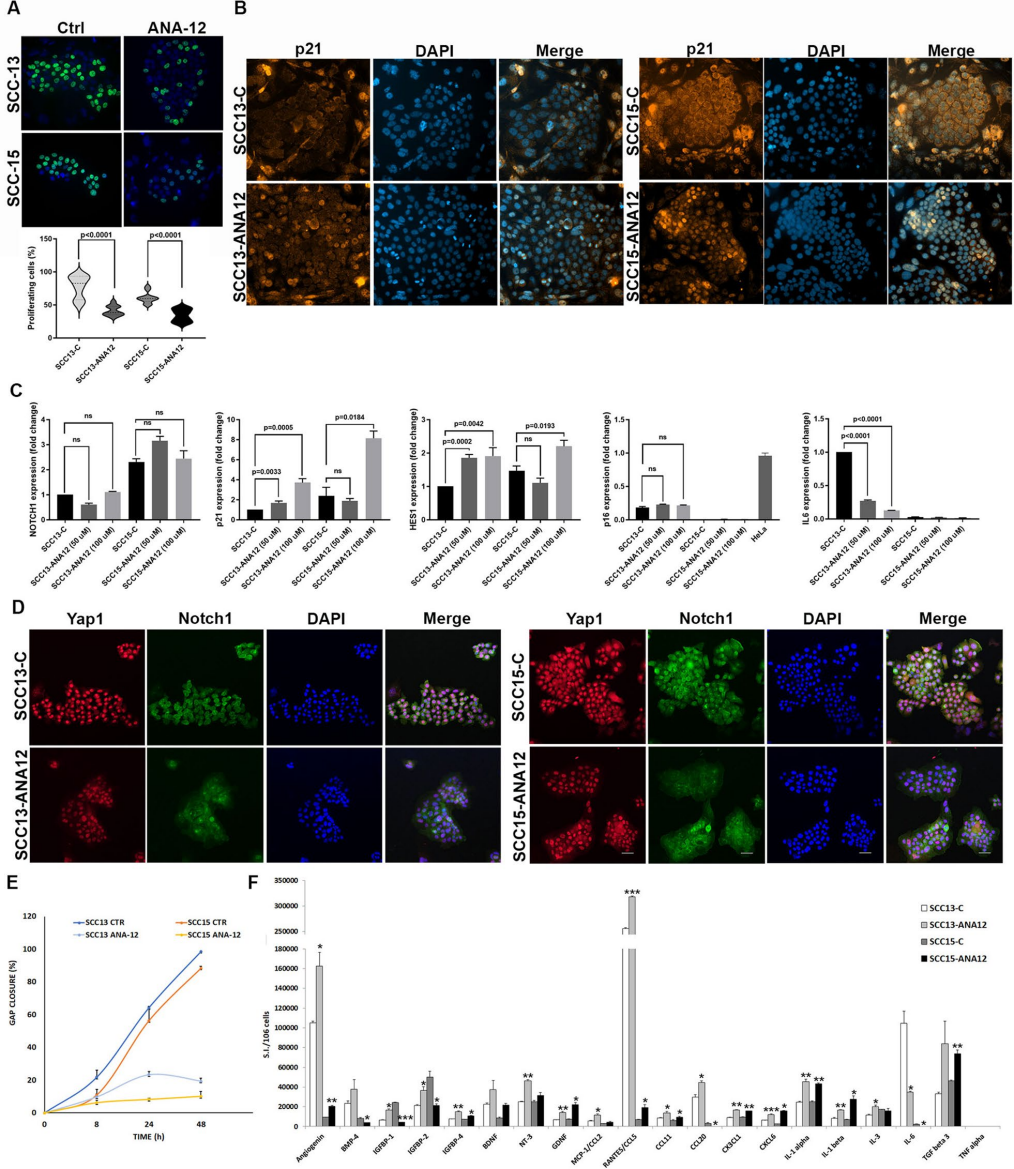
TrkB inhibition induces a tumor-suppressive phenotype in both SCC strains. **A** Representative images of proliferating (green) and total (blue) cells in untreated vs ANA-12-treated SCC cells and percentage values of both SCCs. **B** Representative images of p21 immunostaining (orange) in untreated vs ANA-12-treated SCC cells. DAPI (blue) was used for nuclear staining. **C** Representative images of Yap1 (red) and Notch1 (green) immunostaining in untreated vs ANA-12-treated SCC cells. DAPI (blue) was used for nuclear staining. **D** Fold-change expression of Notch1, p21, Hes1, p16, and IL-6 in untreated vs ANA-12-treated SCC cells by RTqPCR. **E** Values of gap closure of SCC cells against time after treatments. **F** Expression of SASP factors assayed by antibody arrays in supernatants of untreated vs ANA-12-treated SCC cells. Signal intensity (S.I.) is normalized to the cell number. All data are shown as mean ± SD. Significance was determined using a two-tailed Student’s t-test and is indicated in the figure; ns = not significant; *p < 0.05, **p < 0.01, ***p < 0.001. Images were acquired at 20 × magnification for Yap1/Notch1 and 40 × magnification for p21

Fluorescence labeling with 5-ethynyl-2′-deoxyuridine (EdU), a thymidine analog that is incorporated during active DNA synthesis, revealed that ANA-12 treatment reduced the proliferation of SCC cells (Fig. 4A).

In parallel with the reduction of phosphorylated STAT3, ANA-12 treatments induced a significant dose-dependent downregulation of p63 in both SCC lines (Fig. 3D). ANA-12 treatment resulted in Yap1 translocation to the cytoplasm concomitant with decreased nuclear expression of p63 (Supplementary Fig. 5). This effect was more evident in SCC13 cells, which were more morphologically homogeneous and exhibited lower p63 expression levels (Supplementary Fig. 4A). Furthermore, ANA-12 treatment resulted in significant, dose-dependent p21 upregulation (Figs. 3D and 4C) and translocation into the nucleus (Fig. 4B) in both SCC lines. p21 has location-dependent effects, promoting cell survival and proliferation in the cytoplasm while inducing cell-cycle arrest and inhibiting proliferation in the nucleus [61]. Notably, SCC15 cells exhibited higher basal levels of p21 at transcriptional (Fig. 4C) and translational (Fig. 4B and Supplementary Fig. 4A) levels.

Notch signaling is a key mediator of cell–cell communication that controls cell fate decisions, stem cell maintenance, and lineage commitment [37]. E-cadherin–mediated adhesion not only inactivates Yap but also stabilizes and correctly localizes the Notch1 receptor by maintaining epithelial polarity and junctional organization [62]. Close cell–cell contact facilitates interactions between Notch1 and its ligands, enabling effective pathway activation [63]. In epithelial cells, active Notch signaling induces cell-cycle arrest and differentiation and suppresses tumor invasiveness by repressing ΔNp63 and activating genes such as Hes1 and p21 that promote loss of stemness and terminal differentiation [64, 65].

As shown in Fig. 4D, nuclear active Yap is associated with cytoplasmic Notch1 in SCC cells. ANA-12 treatment induces the simultaneous localization of Yap1 in the cytoplasm and Notch1 in the membrane, similar to normal keratinocytes [20]. Notch1 expression is controlled by p53 [61], which is mutated in SCC cells. ANA-12 did not significantly modify Notch1 transcription (Fig. 4C); thus, Notch1 translocation to the membrane was probably due to the recovery of E-cadherin-mediated cell-to-cell contact. Of note, ANA-12-treated cells exhibited a significant increase in the transcription of both p21 and Hes1, which is often used as a readout for Notch activity (Fig. 4C).

Restoring E-cadherin localization and reducing EMT markers promotes a tumor-suppressive, epithelial phenotype characterized by reduced cell migration and invasiveness [51]. Therefore, we investigated the migration capacity of treated SCCs. We monitored the closure of cell-free gaps at 8, 24, and 48 h. ANA-12 treatment reduced the migration of both SCC cell lines compared to untreated cells (Fig. 4E).

Thus, TrkB inhibition induces a tumor-suppressive phenotype in both SCC strains, characterized by reduced proliferation and migration capacity, which is likely due to p63 downregulation, Yap1 inhibition, and Notch1/Hes1 activation.

#### TrkB inhibition and SCC senescence, differentiation, and apoptosis.

In epithelial cancer cells, elevated Notch signaling, marked by increased Hes1 and p21 and reduced p63, indicates a tumor-suppressive shift toward cell-cycle exit and outcomes such as senescence, differentiation, or apoptosis [61, 66].

Senescence is regulated by the CDK inhibitors p21 and p16, with p21 initiating the process and p16 maintaining it [67], and both contribute to downstream effects such as SASP secretion [68]. Here, p16 was barely detectable in both SCC strains, and no increase was observed following ANA-12 administration (Fig. 4C), unlike p21. Secretion of cytokines and growth factors was assessed on SCC culture supernatants following ANA-12 treatments (Fig. 4F). We found a significant increase of several typical SASP proteins (i.e., IL-1α, IL-1β), immune-recruiter or activating chemokines (i.e., CCL2/MCP1, CCL5/RANTES, CCL11, CCL20, CX3CL1, CXCL6), neurotrophic factors (i.e., BDNF, NT-3, GDNF), growth suppression/survival or tissue remodeling mediators (i.e., angiogenin, IGFBP1, IGFBP4, IL3), and proteins that maintain senescence (i.e., TGF-β3, IGFBP2, BMP-4) in SCC13. SCC15 displayed a similar secretion trend compared to SCC13, although IGFBP1/2, IL3, and BMP4 were reduced in SCC15. The key inflammatory cytokine, TNF-α, was absent from the secretome of both SCC lines. TrkB activation promotes IL-6 secretion, establishing a positive inflammatory feedback loop [19]. Notably, the secretion of IL-6 was significantly reduced in both SCCs (Fig. 4F). This finding was confirmed by a significant decrease in IL-6 transcript expression in SCC13 following treatment (Fig. 4C). In SCC15, IL-6 secretion was very low and became barely detectable following ANA-12 administration (Fig. 4C and F).

Basal cells lose their identity and differentiate due to p63 downregulation and Notch1 activation [64, 66]. We observed a reduction in involucrin expression with no significant changes in 14-3-3σ levels (Fig. 3D). The presence of 14-3-3σ may reinforce p21-mediated cell cycle arrest, particularly at the G2/M phase [69]. The decrease in involucrin, despite the upregulation of p21 and Hes1, suggests a partial differentiation state characterized by proliferative arrest without full epithelial maturation. This incomplete differentiation may be due to the absence of secondary signals or persistent repressive chromatin states [64, 66]. Although p21 is usually expressed early in differentiation, sustained overexpression can hinder terminal differentiation by downregulating key markers, such as loricrin and involucrin [70, 71].

Apoptosis is driven by p53 through pro-caspase 3 activation, but its activation can still occur via alternative pathways even when p53 is non-functional. In these contexts, nuclear p21 not only enforces cell-cycle arrest, but it can exert a paradoxical anti-apoptotic effect [72]. Both SCC cells exhibit p53 loss of function [45, 46], and SCC15 cells do not express the protein at all (Fig. 3D, A431 cell line was positive control). After ANA-12 treatment, the expression of both p53 and pro-caspase 3 significantly decreased (Fig. 3D), but the cleaved form of caspase 3 was not present. Interestingly, in treated SCC15 cells, pro-caspase 3 expression even increases. Altogether, these results suggest that both SCC cell types may resist apoptosis.

Thus, TrkB inhibition induces SCC cells to exit the cell cycle, followed by a partial differentiation program and a p21-mediated senescence accompanied by a low inflammatory and moderately immune-activating SASP. Furthermore, TrkB inhibition switches off the feedforward pro-tumorigenic IL6/STAT3 loop.

#### Validation of TrkB blockade in an additional cutaneous SCC model

To further assess the robustness of TrkB-dependent phenotypes across SCC models, key findings were validated in an additional cutaneous SCC cell line.

The cutaneous SCC cell line A431 [29, 73], which harbors a missense *TP53* mutation at codon 273 (c.818G > A; R273H), impairing p53 tumor suppressor function (Supplementary Table 5), expresses BDNF (Supplementary Fig. 3C) and the activated form of TrkB (Supplementary Fig. 6A), and displayed responses to TrkB inhibition comparable to those observed in SCC13 and SCC15 cells, particularly with ANA-12 (100 μM). This treatment significantly reduced STAT3 phosphorylation, in contrast to AKT and ERK signaling, which were not decreased (Supplementary Fig. 6B). These changes were accompanied by decreased expression of the EMT marker Snail, increased E-cadherin levels, and intracellular redistribution of the E-cadherin/Yap1 axis (Supplementary Fig. 6C, D). TrkB inhibition in A431 cells also recapitulated key signaling alterations, including reduced p63 expression, relocalization of p21 and Notch1, increased p21 and Hes1 expression, and was associated with reduced cell proliferation and migration (Supplementary Fig. 6B–G). Finally, IL6 expression was significantly reduced upon ANA-12 treatment of A431 cells (Supplementary Fig. 6H).

#### TrkB inhibition and primary CAF activation

CAFs are major components of the TME that support tumor growth, immune evasion, and therapy resistance [5, 74]. Key regulators drive these tumor-promoting functions: β-catenin drives fibroblast reprogramming and pro-tumorigenic factor expression, Yap1 promotes myofibroblastic activation and matrix remodeling, and Notch1 supports stromal–tumor crosstalk and angiogenic signaling [5, 75–77]. STAT3 signaling acts as a central coordinator of fibroblast activation by stabilizing β-catenin and promoting its nuclear accumulation [78–80]. In turn, Wnt/β-catenin signaling drives Yap1 nuclear translocation [75]. Nuclear Yap1 and Notch1 in CAFs are associated with their activation [5, 81, 82]. To our knowledge, the functional role of TrkB has not yet been studied in CAFs.

**Fig. 5:**
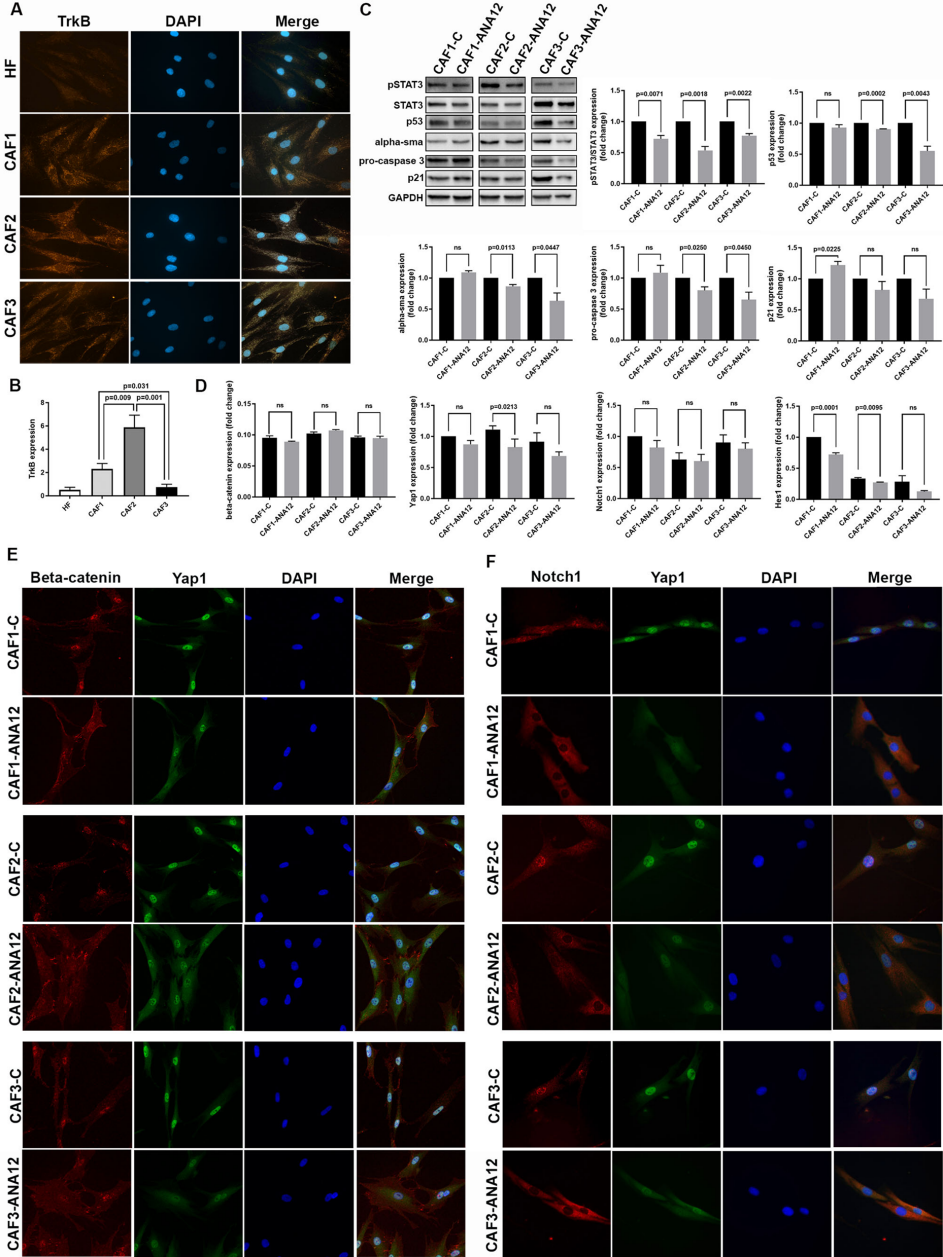
TrkB inhibition dampens key signaling pathways involved in primary CAF activation. **A** Representative images of TrkB immunostaining (orange) in CAFs. DAPI (blue) was used for nuclear staining. **B** Relative TrkB expression in fibroblasts (HF) and CAFs by RTqPCR. **C** Representative immunoblots and related densitometric values (n = 3) of pSTAT3, STAT3, E-cadherin, p53, α-SMA, pro-caspase 3, and p21 in untreated vs ANA-12-treated CAFs. GAPDH was the loading control. **D** Fold-change expression of β-catenin, Yap1, Notch1, and Hes1 in untreated vs ANA-12-treated CAFs by RTqPCR. **E** Representative images of β-catenin (red) and Yap1 (green) immunostaining in untreated vs ANA-12-treated CAFs. **F** Representative images of Notch1 (red) and Yap1 (green) immunostaining in untreated vs ANA-12-treated CAFs. DAPI (blue) was used for nuclear staining. All data are shown as mean ± SD. Significance was determined using a two-tailed Student’s t-test and is indicated in the figure; ns = not significant. Images were acquired at 20 × magnification

We previously isolated and characterized three primary CAF cultures from SCCs of patients [20] (Supplementary Table 6). All CAF strains expressed the TrkB protein and its phosphorylated form (Fig. 5A and Supplementary Fig. 3D), which are primarily localized in the cytoplasm, with interindividual variability. In some cells, p-TrkB staining is mainly perinuclear. CAF2 exhibited higher TrkB expression than the other cell strains at both the transcriptional and translational levels (Fig. 5A and B). CAFs were also able to express BDNF (Supplementary Fig. 3C).

Despite interindividual differences, ANA-12 treatment significantly inhibited the phosphorylation of the transcription factor STAT3 in all CAF strains (Fig. 5C). No consistent reduction of pERK/ERK or pAKT/AKT ratios was observed (Supplementary Fig. 4D). No significant changes in β-catenin transcription were observed following ANA-12 treatment (Fig. 5D). A trend of reduced Yap1 transcription was found in all ANA-12-treated strains (Fig. 5D). Notably, all CAF strains exhibit nuclear expression of β-catenin and Yap1 in most cells, and their expression shifts after ANA-12 administration (Fig. 5E). ANA-12-treated CAFs exhibit β-catenin localized at the membrane, accompanied by increased Yap1 expression in the cytoplasm (Fig. 5E). All CAF strains displayed nuclear expression of both proteins. After ANA-12 treatment, they translocated to the cytoplasm (Fig. 5F). Notch transcript expression did not vary (Fig. 5D), whereas Hes1 expression was significantly reduced, indicating Notch1 pathway inhibition (Fig. 5D).

Thus, TrkB inhibition dampens key signaling pathways involved in CAF activation, such as the β-catenin, Yap1, and Notch1 pathways.

#### TrkB inhibition and primary CAF reprogramming

Simultaneously suppressing the activity of the β-catenin, YAP1, and Notch1 pathways in CAFs can significantly reprogram the TME toward a less supportive or even tumor-suppressive state [75, 76].

**Fig. 6:**
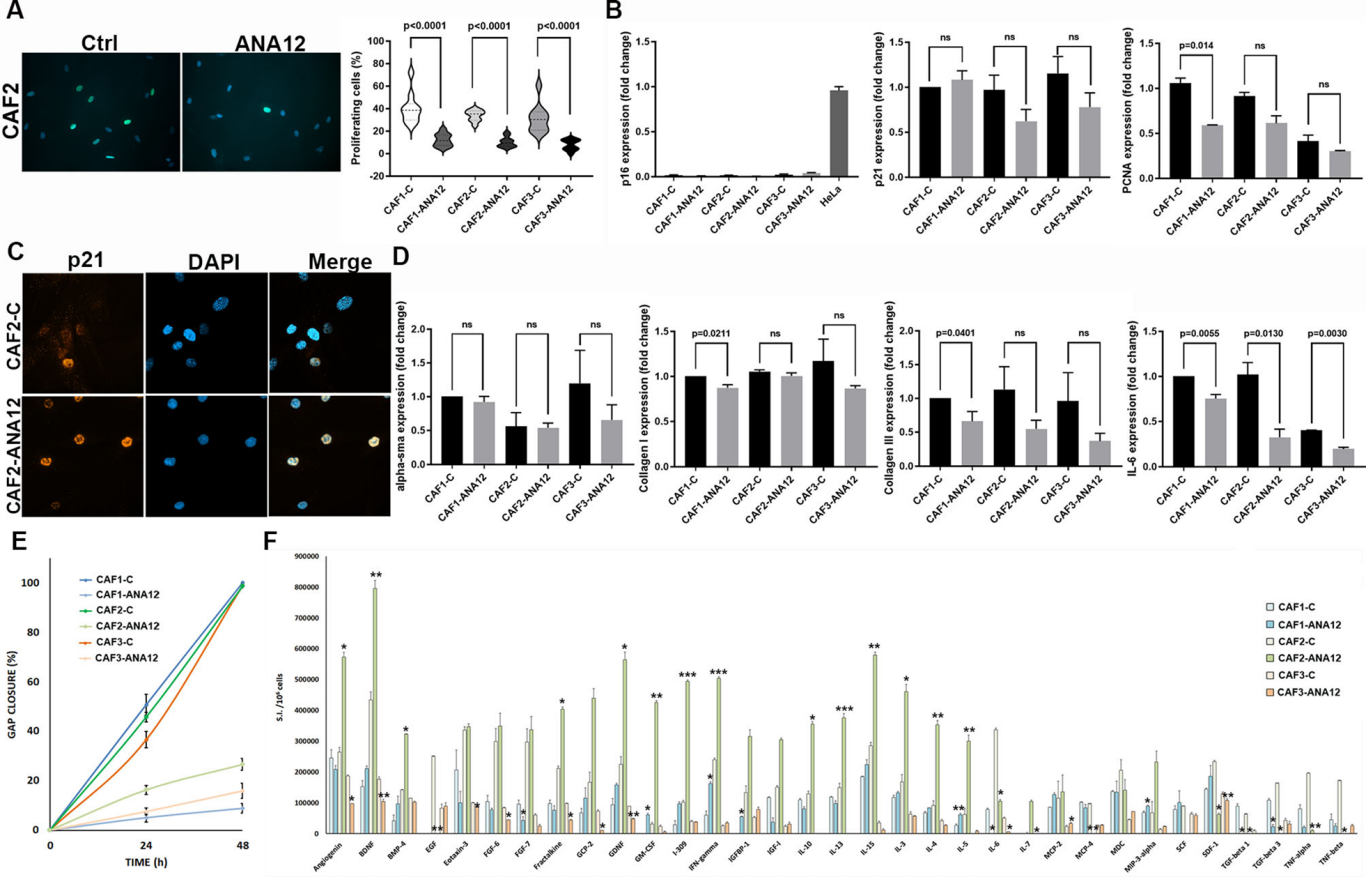
TrkB inhibition induces a tumor-non-supportive state in primary CAFs. **A** Representative images of proliferating (green) and total (blue) cells in untreated vs ANA-12-treated CAFs and percentage values of all strains. **B** Fold-change expression of p16, p21, and PCNA in untreated vs ANA-12-treated CAFs by RTqPCR. **C** Representative images of p21 immunostaining (orange) in untreated vs ANA-12-treated CAFs. DAPI (blue) was used for nuclear staining. **D** Fold-change expression of α-SMA, collagen I, collagen III, and IL-6 in untreated vs ANA-12-treated CAFs by RTqPCR. **E** Values of gap closure of CAFs against time after treatments. **F** Expression of SASP factors assayed by antibody arrays in supernatants of untreated vs ANA-12-treated CAFs. Signal intensity (S.I.) is normalized to the cell number. All data are shown as mean ± SD. Significance was determined using a two-tailed Student’s t-test and is indicated in the figure; ns = not significant; *p < 0.05, **p < 0.01, ***p < 0.001. Images were acquired at 20x (EdU assay) and 40x (p21 expression) magnification

The EdU assay revealed that ANA-12 treatment reduced the proliferation of all CAF strains (Fig. 6A). Considering proteins that regulate the cell cycle, p53 expression decreased (Fig. 5C), while p16 expression remained unchanged following ANA-12 treatment (Fig. 6B). p21 protein levels increased only in CAF1 cells. In the other strains, a reduction trend was observed (Fig. 5C). p21 transcripts followed a similar pattern (Fig. 6B). p21 was expressed in the nucleus of some CAF cells, and ANA-12 treatments increased the percentage of nuclear-positive cells, suggesting an effect on the cell cycle (Fig. 6C). Notably, ANA-12 treatment downregulates PCNA (Fig. 6B), which significantly impacts the cell cycle by impairing DNA replication and S-phase progression [83]. Furthermore, pro-caspase 3 levels decreased with ANA-12 treatment (Fig. 5C) without an increase in its cleaved form, suggesting protection from apoptosis.

Among the three CAF strains, CAF2 exhibits a more fibrotic phenotype (Supplementary Fig. 4). ANA-12 administration decreased the expression of α-smooth muscle actin (α-SMA), a fibrotic marker, in CAF2 and CAF3 (Fig. 5C). α-SMA transcripts did not vary significantly (Fig. 6D). Drug treatments significantly reduced collagen I and III expression in CAF1 (Fig. 6D). A trend of collagen III reduction was also observed in CAF2 and CAF3.

To investigate CAF migration, we monitored the closure of cell-free gaps at 24 and 48 h. ANA-12 treatment reduced the migration of all CAF strains compared to untreated cells (Fig. 6E).

SASP mediators were expressed at higher levels in CAFs than in SCC cell lines. However, the three CAF strains exhibited distinct basal levels of secreted proteins. Despite interindividual variability, ANA-12 treatment modulated their CAF secretome (Fig. 6F). Treated CAF1 cells displayed the up-regulation of SASP effectors that promote immune activation and recruit cytotoxic and effector immune cells (i.e., CXCL6, GM-CSF, IL-3, IL-15, IFN-γ, I-309, MIP-3-α, IGFBP-1, SCF, SDF-1), accompanied by downregulation of proteins that suppress immunity and support tumor growth, angiogenesis, and immune evasion (i.e., IL-6, IL-10, IL-13, TGF-β1/3, TNF-α/β, IGF-I, CX3CL1, Angiogenin, FGF-6, FGF-7). CAF2 was the strain with higher levels of secreted protein. ANA-12 treatments increased a broad mix of growth factors, neurotrophins, cytokines, and chemokines, some of which are pro-regenerative, pro-angiogenic, or immunoregulatory (i.e., Angiogenin, BDNF, BMP-4, FGF-6, FGF-7, Fractalkine, GCP-2, GDNF, GM-CSF, I-309, IFN-gamma, IGFBP-1, IGF-I, IL-10, IL-13, IL-15, IL-3, IL-4, IL-7, MCP-2, MIP-3-α), alongside a decrease of well-known pro-inflammatory, pro-fibrotic, and immunosuppressive factors (i.e., IL-6, MCP-4, MDC, SCF, SDF-1, TGF-β 1, TGF-β 3, TNF-α, TNF-β). CAF3 exhibited a narrow, selective modulation of CAF-secreted proteins with a mix of pro-inflammatory, growth-related, and chemokine components (i.e., IFN-gamma, IGFBP-1, IGF-I, IL-5, MCP-2, MDC, MIP-3-α), along with the downregulation of several growth, neurotrophic, and immunoregulatory signals (i.e., Angiogenin, BDNF, BMP-4, Eotaxin-3, FGF-6, FGF-7, Fractalkine, GCP-2, GDNF, GM-CSF, IL-15, IL-3, IL-4, IL-6, SDF-1, TGF-β 1, TGF-β 3, TNF-β). Notably, the secretion of IL-6 was significantly reduced in all CAF strains. This finding was confirmed by the reduction in IL-6 transcript expression after treatment (Fig. 6D).

Thus, TrkB inhibition orchestrates a coordinated and multitarget reversion of CAFs from an activated, tumor-supportive state to a quiescent and phenotypically normalized one by suppressing proliferative, migratory, fibrotic, and inflammatory pathways.

#### TrkB inhibition in 3D SCC models

Solid tumors grow in a 3D structure, where cells interact with each other and the ECM, and are exposed to diverse environmental biochemical gradients (e.g., oxygen, growth factors, nutrients). Thus, 3D cultures more accurately replicate the in vivo situation by preserving the 3D architecture of tumors [84]. Human cSCC organotypic cultures simulate the in vivo epidermis and dermis, including cellular interactions and metabolic properties. These models provide mechanistic insights into tumor progression and enable the validation of potential therapeutic targets [20, 85–87].

**Fig. 7:**
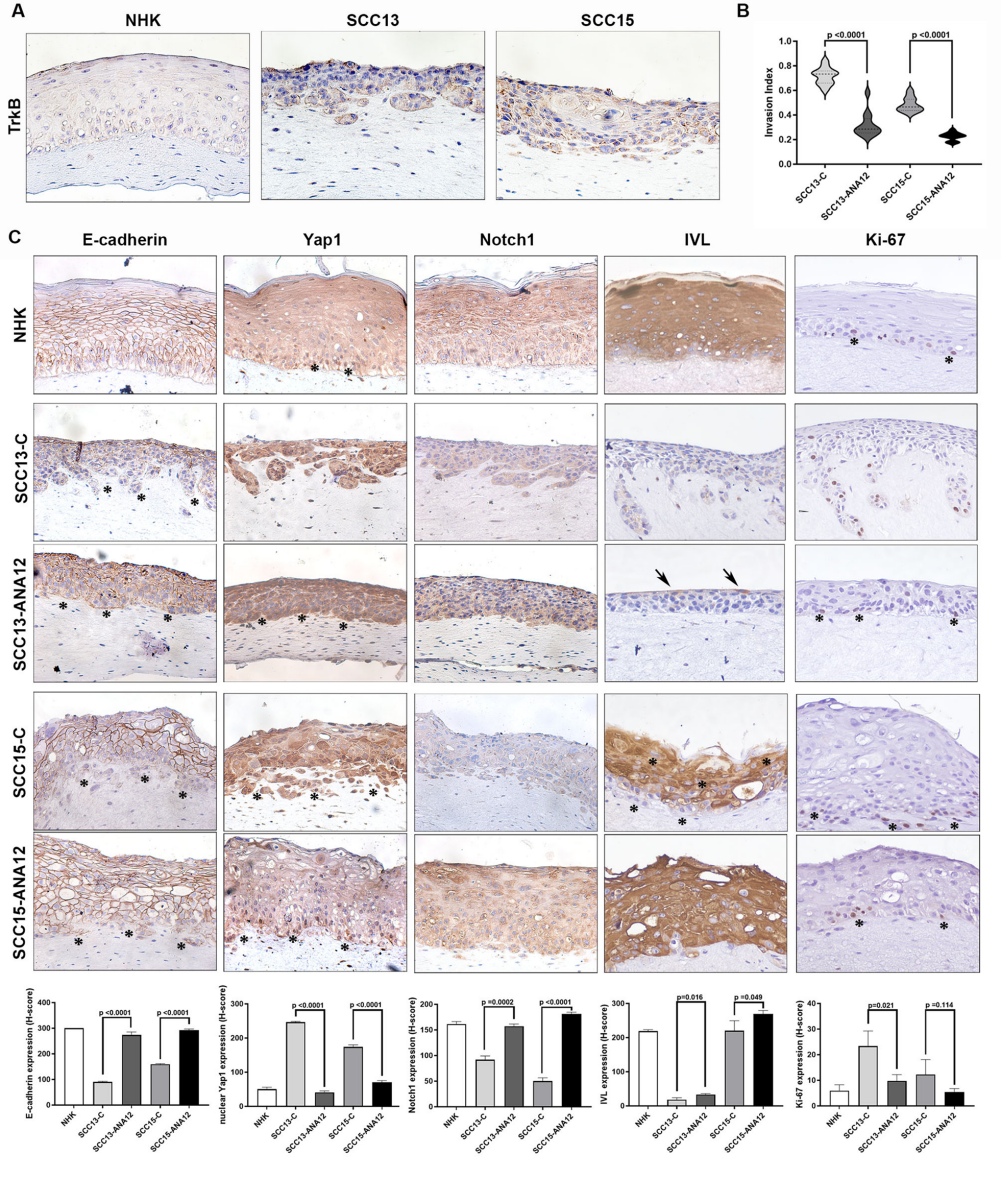
TrkB inhibition induces a tumor-suppressive phenotype in both SCC strains in 3D organotypic models. **A** Representative images of TrkB immunostaining of 3D organotypic models generated by seeding primary normal human keratinocytes (NHK) or SCC cells on a matrix incorporating human dermal fibroblasts or CAFs. **B** Invasion Index values of untreated vs ANA-12-treated 3D SCC models (n = 10 fields). **C** Representative images of paraffin-embedded specimens of NHK and untreated vs ANA-12-treated SCC 3D models immunostained with antibodies against E-cadherin, Yap1, Notch1, involucrin (IVL), and Ki-67. The histological index (H-index) of each specimen was calculated. All data are shown as mean ± SD. Significance was determined using a two-tailed Student’s t-test and is indicated in the figure. Images were acquired at 20 × magnification

SCC13 and SCC15 lines were grown in a 3D organotypic model characterized by a similar dermal matrix containing CAFs. As shown in Fig. 7A, 3D SCC models exhibited cellular invasion and TrkB expression similar to SCCs from patients (Fig. 1).

ANA-12 treatment significantly reduced the invasive ability of SCC cells (Fig. 7B) by modulating specific pathways (Fig. 7C and Supplementary Fig. 7). In 3D models generated using primary keratinocytes (NHK) and fibroblasts, E-cadherin was localized to cell–cell adhesion membranes, whereas Yap1 was mainly cytoplasmic in differentiated keratinocytes and nuclear in some basal cells (asterisks). Notch1, involucrin, and 14–3-3σ were highly expressed in differentiated cells. The percentage of cells expressing Ki-67, a nuclear marker of proliferating cells, was very low (asterisks). Nuclear p63 expression was higher in basal and suprabasal layers (asterisks), resembling normal skin staining patterns [88].

The SCC13 model exhibited a poorly differentiated and highly invasive phenotype. Compared to the 3D skin model, it showed reduced E-cadherin membrane localization, especially in basal cells (asterisks), elevated Yap1 in the cytoplasm and nucleus, decreased Notch1, barely detectable involucrin and 14-3-3σ expression, increased Ki-67-positive nuclei in invasive cells, and widespread p63 expression. ANA-12 treatment restored E-cadherin membrane localization (including in basal cells, asterisks), decreased nuclear Yap1 (asterisks), and increased cytoplasmic Yap1 and Notch1 expression in most cells. Involucrin expression was evident in the outermost layer of keratinocytes (arrows), though 14-3-3σ expression remained unchanged. ANA-12-treated SCC13 models also displayed reduced Ki-67-positive nuclei and decreased p63 staining. More positive nuclei are mostly restricted to basal cells (asterisks).

The SCC15 model, which had lower invasive potential, showed decreased E-cadherin membrane localization in basal and invasive cells (asterisks), increased Yap1, which displayed more nuclear expression in the nucleus of these cells (asterisks), and reduced Notch1, compared to the 3D skin models. Although SCC15 cells could differentiate and express involucrin, differentiation appeared incomplete, as evidenced by negative staining in some suprabasal cells (asterisks), unlike in 3D skin models. Decreased expression of 14-3-3σ was also noted (asterisks). Notably, invasive cells were involucrin- and 14-3-3σ-negative. Ki-67 was mainly expressed in invasive cells (asterisks), and p63 was expressed in most nuclei. ANA-12 treatment increased E-cadherin membrane localization in basal keratinocytes (asterisks), reduced nuclear Yap1, especially in basal cells (asterisks). It also increased Notch1, involucrin, and 14-3-3σ (asterisks) expression. ANA-12-treated SCC15 showed fewer Ki-67-positive nuclei and decreased p63 staining, with nuclear positivity primarily in basal cells (asterisks).

Thus, TrkB inhibition reduces SCC cell proliferation and invasion abilities, as well as induces the expression of certain differentiation markers, mainly in SCC15 cells in 3D models. ANA-12 treatment also restores cell-to-cell adhesion, dampens Yap1 and p63 signaling, and induces Notch1 in SCC cells. Data were consistent with a tumor-suppressive and pro-differentiative effect.

## Discussion

The TrkB axis is a critical signaling pathway that promotes EMT, as well as the migration and invasion of epithelial malignancies [14–18]. However, its role in cSCCs remains poorly understood.

We previously demonstrated that TrkB activation drives age-associated keratinocyte plasticity and preneoplastic changes [20]. As it represents a crucial event in cSCC development, TrkB could be considered a potential target for therapeutic intervention. Our present study indicates that activation of the TrkB pathway, evidenced by p-TrkB expression in tumor keratinocytes and mesenchymal cells along with BDNF presence, may contribute to tumor–stroma interactions and support SCC progression.

Here, we demonstrate that:

i) the *‘TrkB, E-cadherin, Yap1, Notch1’* signature can serve as a clinically meaningful biomarker for both cSCC subtype classification and identification of high-risk cases;
ii) TrkB blockade reprograms SCC cells toward a tumor-suppressive state, by simultaneously targeting proliferative, EMT, stemness, and differentiation pathways, thereby promoting tissue homeostasis in both 2D and 3D models;
iii) TrkB blockade reprograms CAFs toward a less inflammatory, less proliferative, and less fibrotic phenotype by simultaneously suppressing key activating signaling pathways.

### A TrkB-based signature as a relevant biomarker for cSCC and prediction models

Risk stratification in cSCC is critical for clinical decision-making and outcome prediction. While conventional histopathologic grading remains useful, it often lacks sensitivity in identifying aggressive tumors, especially in early or ambiguous cases [43, 89]. IHC-based signatures represent a valuable complementary approach, given their accessibility, cost-effectiveness, and ease of integration into routine diagnostics [90, 91]. Such panels may enable more accurate patient risk stratification, supporting tailored treatment and surveillance strategies, reducing overtreatment of low-risk cases, and ensuring appropriate management of high-risk disease [92, 93].

Based on differential expression and ROC analysis, we found that the specific *‘TrkB, E-cadherin, Yap1, Notch1’* signature can represent a clinically relevant biomarker for both distinguishing cSCC subtypes and identifying high-risk cases. Our study demonstrates that machine learning can be effectively applied to classify cSCC types as well as cSCC risk based on IHC-biomarker expression, even when working with a limited dataset. Beyond classification accuracy and prediction, machine learning offers a structured way to explore the underlying patterns in the data, revealing which biomarkers carry the most discriminative weight. Our analysis highlights key features, such as TrkB, Yap1, and Notch1, as consistently informative, and also emphasizes the added value of including stromal markers. Overall, the developed workflow serves as a prototype for a prediction model. If more data becomes available, the same structure can be scaled up, and performance metrics can be recalibrated with greater confidence for clinically reliable classifiers.

Although RF sometimes shows slightly higher variability across folds compared to LR, it generally maintains balanced precision and recall, which is crucial for small or imbalanced datasets. Its ability to identify meaningful features in addition to predicting class labels makes it particularly valuable for understanding underlying biological patterns. Furthermore, RF performance remains stable when stromal features were included, indicating robustness to heterogeneous data.

Overall, although RF does not always achieve the absolute highest CV accuracy, it combines solid predictive performance with interpretability and resilience to small, complex datasets. Thus, it represents the most reliable classifier among those tested in our study. LR, which often matches or exceeds RF in accuracy, can be considered a complementary baseline, offering additional stability in scenarios with very limited samples.

### TrkB blockade in SCC cells

Although immune checkpoint inhibitors represent a major advance in the treatment of advanced cSCC, their use is limited in immunocompromised patients due to toxicity, resistance, and accessibility issues [1, 2]. EGFR inhibitors, particularly cetuximab, offer an alternative for patients unsuitable for immunotherapy, but modest response rates and acquired resistance restrict their effectiveness [1, 2]. Thus, alternative or multimodal strategies remain crucial for patients who are ineligible for immunotherapy or do not respond to targeted therapies. Interestingly, ERK, PI3K/AKT, and STAT3 pathways play key roles in resistance to both EGFR-targeted therapies and chemotherapeutics [17].

Our study demonstrates that TrkB blockade modulates key signaling pathways involved in epithelial cell proliferation, differentiation, and senescence in SCC cells, in line with a tumor-suppressive and differentiation-promoting effect. Consistent with the typical genetic profile of SCC, SCC13, SCC15, and A431 cell lines exhibit *TP53* loss-of-function mutations [45, 46], which lead to impaired p53 tumor suppressor activity. The TrkB antagonist ANA-12 significantly reduces STAT3 phosphorylation and shows a trend toward decreased pERK/ERK ratio, accompanied by reduced IL-6 expression. This coordinated modulation is consistent with the observed inhibition of proliferation and indicates the disruption of the pro-tumorigenic IL-6/STAT3 feedback loop, commonly found in epithelial tumors. Indeed, STAT3 not only responds to IL-6, but it can also drive its expression (19). Since IL-6/STAT3 signaling is frequently upregulated in mutant *TP53* tumors and contributes to immune evasion and cancer cell plasticity [19, 94], its inhibition may further reinforce the anti-tumor and anti-EMT effects of ANA-12.

The reversal of EMT, evidenced by decreased expression of p63 and EMT transcription factors alongside upregulation of E-cadherin and re-establishment of cell–cell-adhesion, indicates that ANA-12 restores epithelial cell identity and reduces migration and invasiveness [95]. This EMT reversal is further supported by the cytoplasmic localization of Yap1, which is consistent with Hippo pathway activation. Reduced nuclear Yap1 levels decrease the transcriptional programs associated with cell proliferation, stemness and plasticity [96, 97], thereby reinforcing the ANA-12 anti-proliferative and anti-migratory effects.

ANA-12 simultaneously activates Notch signaling, as evidenced by the upregulation of Hes1 expression. Notch signaling exerts context-dependent effects in epithelial tissues, promoting either progenitor maintenance or terminal differentiation [37]. Here, G1 cell cycle arrest is accompanied by a significant p63 decrease, Yap1 cytoplasmic retention, Notch1 activation, and p21 increase, independently of wild-type p53 activity. Therefore, cell cycle exit and differentiation are likely mediated via p53-independent pathways, such as Notch1, p63, and Yap1 crosstalk [61, 98]. The downregulation of p63, coupled with increased involucrin expression in 3D cultures, suggests a shift from stem-like to differentiated keratinocyte states, which promotes terminal differentiation, primarily in SCC15 cells. Interestingly, ANA-12 induced involucrin and 14-3-3σ expression in the 3D culture system, but not in 2D. This suggests that terminal differentiation is re-engaged only in physiologically relevant contexts. For example, Yap activity is sensitive to cell density and mechanical tension [98], and its inactivation in the presence of active Notch signaling may shift the balance toward terminal differentiation in a 3D environment. These differential behaviors highlight the importance of 3D architecture (e.g., stratification and cellular polarity), mechanical cues, and microenvironmental signals in enabling the full activation of terminal differentiation programs, which may be limited in 2D models.

Caspase 3 remained unchanged, indicating that apoptosis is not a primary mechanism of ANA-12 action in this context, further emphasizing a senescence-driven growth arrest. Interestingly, ANA-12 triggers a SASP characterized by increased secretion of a few inflammatory cytokines and some chemokines that can recruit immune cells. The major proinflammatory cytokine TNF-α is absent, and IL-6 is reduced. Additionally, neurotrophic factors, growth modulators, and tissue remodeling proteins, as well as senescence stabilizers, are increased. Thus, the ANA-12 secretome exhibits a SASP profile that favors immune recruitment and tissue remodeling instead of overt inflammatory cytotoxicity [68, 99]. These proteins likely reinforce senescence-associated growth arrest, modulate the tumor microenvironment, promoting immune surveillance and tissue homeostasis [67].

Thus, ANA-12 promotes a non-lethal reprogramming of epithelial cells, favoring epithelial maturation and homeostasis, particularly under physiologically relevant 3D conditions. Taken together, our data indicate a promising therapeutic approach for reprogramming SCC cells with mutant *TP53*, which is often associated with therapy resistance, toward a less aggressive, more differentiated, and immunomodulatory state.

### TrkB blockade in primary CAFs

CAFs may contribute to resistance to checkpoint immunotherapy, suggesting that targeting them could improve response rates. However, effective clinical approaches for targeting CAFs are lacking [100]. Phenotypic and functional heterogeneity is a prominent characteristic of CAFs, as revealed by transcriptomic analyses of distinct CAF subpopulations [5]. Thus, identifying specific, common networks that govern their activation could be a significant advancement for CAF-targeted therapies.

Our study demonstrates that ANA-12 suppresses multiple signaling pathways, including IL-6/STAT3, Notch/Hes1, and Wnt/β-catenin/Yap1, in a coordinated manner to remodel CAFs toward a non-inflammatory, non-fibrotic, non-migratory, and non-proliferative state, regardless of the analyzed strain.

STAT3 is a key driver of CAF activation and interaction with tumor and immune cells within the TME through the induction of pro-tumorigenic secreted proteins, including IL-6, VEGF, and TGF-β. Notably, IL-6 is a target of both β-catenin and Notch1 in CAFs [3, 4]. Consistently, STAT3 activation correlates with tumor progression, immune suppression, metastasis, and therapy resistance [101, 102], whereas its inactivation shifts stromal cells toward a more adherent, less migratory phenotype [5]. By consistently reducing STAT3 phosphorylation and IL-6 levels across CAF strains, ANA-12 can disrupt this pro-tumorigenic feedback loop, thereby reducing proliferation, migration, and suppressing CAF-driven tumor support. Increased nuclear p21, cytoplasmic sequestration of Yap1, reduced PCNA, IL-6, and Hes1 levels, which can induce the cyclin-dependent kinase inhibitor p27^Kip1^, can collectively contribute to G1 cell cycle arrest. The absence of p16 or p53 induction alongside stable caspase 3 and modulated SASP suggests the presence of p21-mediated senescence rather than full senescence or apoptosis.

Recent findings suggest that targeting myofibroblastic CAFs could be a promising strategy for converting immune cold tumors into more responsive ones [100]. Simultaneous activation of the Wnt/β-catenin and Yap1 pathways is necessary to induce the CAF pro-tumoral phenotype and ECM remodeling [75]. Similarly, Notch signaling plays a crucial role in maintaining the myofibroblast-like and ECM-producing phenotype of CAFs [44, 103]. By inducing β-catenin relocalization to the membrane and facilitating Yap1 cytoplasmic retention, ANA-12 can concurrently suppress both Wnt-driven transcription of proliferation and survival genes and Yap1-mediated expression of ECM-related and proliferative targets and the pro-fibrotic program. The observed cytoplasmic shift of Notch1 and the downregulation of Hes1 following ANA-12 treatment are consistent with a Notch-inhibitory mechanism. Thus, inhibition of these three pathways can steer CAFs away from their activated state. ANA-12 significantly reduced α-SMA protein expression in CAF2, which is the more fibrotic strain, and CAF3. On the other hand, CAF1 exhibited a significant collagen I decrease. A trend of collagen III reduction was found in all CAF strains. Notably, ANA-12 modulates the Notch1 pathway differently in SCC cells and CAFs, suggesting that it could be a safer alternative to direct Notch inhibitors. While Notch inhibitors have anti-fibrotic effects, they can also promote tumor growth in epithelial cells due to Notch1 dual role [34].

CAFs are heterogeneous in origin and function, resulting in variable SASP profiles [95]. In all CAF strains, ANA-12 reduces key immunosuppressive mediators (e.g., IL-6, TGF-β1/3, TNF-α/β) and enhances immune-recruiting signals (e.g., IFN-γ, IL-15, MIP-3α), suggesting that it can reprogram activated CAFs toward a pro-immunogenic phenotype, which could restore anti-tumor immune surveillance. Notably, the differential ANA-12-mediated modulation of the SASP, observed across CAF1–3, underscores their functional heterogeneity and supports the concept of subtype-specific plasticity [5]. CAF1 exhibits the most significant shift in the SASP toward immune stimulation, characterized by the upregulation of chemokines and cytokines that promote the recruitment and activation of cytotoxic immune cells, alongside the downregulation of pro-tumoral and immunosuppressive proteins. CAF2, which displayed the highest basal SASP activity, exhibits broader and more complex reprogramming with ANA-12 treatment. Several pro-regenerative and immunomodulatory proteins are upregulated while key pro-fibrotic and inflammatory mediators are reduced. CAF3 exhibits the narrowest SASP modulation, marked by the selective upregulation of immune- and growth-related factors and the downregulation of growth and neurotrophic signals. Despite the limited breadth of changes, the consistent reduction in immunosuppressive cytokines (e.g., IL-6, TGF-β1/3, and TNF-β) aligns with the broader immunomodulatory effects observed in CAF1 and CAF2.

Taken together, our findings indicate that ANA-12 treatment can be a novel approach to modulating primary CAF function, reprogramming them toward a non-tumor-supportive state. Tumor-stroma reprogramming strategies that aim to normalize, rather than eliminate, the activated cells may preserve stromal integrity, avoiding permanent tissue damage.

## Conclusions

Our findings provide a basis for improved high-risk patient stratification by highlighting a TrkB-based signature and generating a prototype predictive model with potential prognostic value.

Furthermore, TrkB was found to be a multifaceted therapeutic target. ANA-12 is still in the experimental phase and not yet in clinical use; however, its effectiveness in SCC models supports exploring TrkB inhibition as a therapeutic strategy in preclinical models. Targeting TrkB-mediated signaling, particularly the downstream STAT3 pathways, could make cSCCs more susceptible to chemo- or targeted therapies and overcome therapeutic resistance. Furthermore, ANA-12 deactivates CAFs, converting them to a non-tumor-supportive state, and may rewire the microenvironment to favor anti-tumor immunity with resolution of inflammation. These insights support the potential of combining TrkB inhibition with immunotherapeutic strategies.

Overall, our findings open avenues for patient stratification and combining targeted therapeutic development.

## Supplementary Information

Additional file1 (DOCX 50 KB)

Additional file2 (DOCX 2697 KB)

Additional file3 (PDF 777 KB)

## Data Availability

All data generated or analysed during this study are included in this published article and its supplementary information files.

## List of abbreviations

cSCC: Cutaneous Squamous Cell Cancer
TME: Tumor immune microenvironment
EMT: Epithelial-mesenchymal transition
ECM: Extracellular matrix
CAFs: Cancer-associated fibroblasts
TrkB: Tropomyosin receptor kinase B
YAP1: Yes-associated protein 1
HES1: Hairy and Enhancer of Split-1
STAT3: Signal Transducer and Activator of Transcription 3
IL-6: Interleukin-6
ERK: Extracellular signal-Regulated Kinase
AKT: AKR Thymoma-derived oncogene
CDKI: Cyclin-Dependent Kinase Inhibitor
