# Supplementary material for: TRKB-based signature identifies high-risk squamous cell carcinoma cases and TRKB blockade reprograms tumor and stromal cells toward suppressive phenotypes
Source: J Biomed Sci. 2026 Feb 25;33:22. doi: 10.1186/s12929-026-01227-0 (PMC12934051; doi:10.1186/s12929-026-01227-0)
Supplement: Supplementary file 1 — Additional file1 (DOCX 50 KB) [file 12929_2026_1227_MOESM1_ESM.docx]

**Supplementary Tables**

**Supplementary Table 1. Primer sequences for the genes assessed using qRT-PCR**

| ***mRNA*** | ***Forward*** | ***Reverse*** |
| --- | --- | --- |
| ***TrkB*** | *5’ AGGAAATTCACGACGGAAAG 3’* | *5’ TCACCTCATTGTTTGACAGC 3’* |
| ***Slug*** | *5’GACACGGCGGTCCCTACAG 3’* | *5’AGGAGCCGGGTGACTTCAGA 3’* |
| ***Twist*** | *5’CTCAGCTACGCCTTCTCGGT 3’* | *5’ATCTAGGTCTCCGGCCCTGC 3’* |
| ***E-cadherin*** | *5’AGTTTTCCACCAAAGTCACGC 3’* | *5’AGGAGTTGGGAAATGTGAGCA 3’* |
| ***β-catenin*** | *5’AGTGCTGAAGGTGCTATCTGT 3’* | *5’TGGATCTGTCAGGTGAAGTCC 3’* |
| ***Yap1*** | *5’AACTGCTTCGGCAGGCAATG 3’* | *5’GAGTCCCACCATCCTGCTCC 3’* |
| ***Notch1*** | *5’GGCCACCCCTCCTAGTTTGG 3’* | *5’TCCCTCACTGGCATGACACA 3’* |
| ***Hes1*** | *5’ATGACAGTGAAGCACCTCCG 3’* | *5’CGTTCATGCACTCGCTGAAG 3’* |
| ***p21^Waf1^*** | *5’TGTCTTGTACCCTTGTGCCTC 3’* | *5’GAGAAGATCAGCCGGCGTTT 3’* |
| ***p16*** | *5’GCCCGGGGTCGGGTA 3’* | *5’TCATCATGACCTGGATCGGC 3’* |
| ***PCNA*** | *5’CAAGTAATGTCGATAAAGAGGAGG 3’* | *5’GTGTCACCGTTGAAGAGAGTGG 3’* |
| ***Alpha-sma*** | *5’CTGACCCTGAAGTACCCG 3’* | *5’AGTGAGATCTCGGCCAGC 3’* |
| ***Collagen I*** | *5’CTGCTGGACGTCCTGGTGAA 3’* | *5’ACGCTGTCCAGCAATACCTTGAG 3’* |
| ***Collagen III*** | *5’TCCAACTGCTCCTACTCG 3’* | *5’CGAGTCCTCCTACTGCTA 3’* |
| ***IL6*** | *5’CAATCTGGATTCAATGAGGAGAC 3’* | *5’CTCTGGCTTGTTCCTCACTACTC 3’* |
| ***BDNF*** | *5’GACATCATTGGCTGACACTTT 3’* | *5’TACTGAGCATCACCCTGGAC 3’* |
| ***GUSB*** | *5’TGCAGGTGATGGAAGAAGTG 3’* | *5’ TTGCTCACAAAGGTCACAGG 3’* |

**Supplementary Table 2. Antibodies for immunoblot, immunofluorescence (IF), and immunohistochemistry (IHC)**

| **Antibody** | **Company** | **Catalog Number** | **Description** | **Immunoblot** | **IF** | **IHC** |
| --- | --- | --- | --- | --- | --- | --- |
| ***TrkB*** | Santa Cruz, Dallas, Texas, USA | SC-377218 | Mouse monoclonal |  | 1:100 | 1:200 |
| ***Phospho-TrkB*** | Thermo Fischer Scientific, Waltham, MA, USA | PA5-36695 | Rabbit polyclonal |  | 1:1000 | 1:200 |
| ***E-cadherin*** | BD Biosciences, San Jose, CA, USA | 610181 | Mouse monoclonal | 1:2000 | 1:1000 |  |
| ***E-cadherin*** | DAKO, Agilent | M3612 | Mouse monoclonal |  |  | 1:100 |
| ***β-catenin*** | Santa Cruz, Dallas, Texas, USA | SC-7963 | Mouse monoclonal |  | 1:200 |  |
| ***Notch 1*** | Abcam, Cambridge, UK | 65297 | Rabbit polyclonal |  | 1:200 |  |
| ***Notch 1*** | Santa Cruz, Dallas, Texas, USA | SC-376403 | Mouse monoclonal |  |  | 1:100/1:50 |
| ***BDNF*** | Abcam, Cambridge, UK | ab203573 | Mouse monoclonal |  |  | 1:100 |
| ***Yap1*** | Santa Cruz, Dallas, Texas, USA | SC-101199 | Mouse monoclonal |  | 1:200 |  |
| ***Yap1*** | Santa Cruz, Dallas, Texas, USA | SC-376830 | Mouse monoclonal |  |  | 1:200 |
| ***Yap1*** | Santa Cruz, Dallas, Texas, USA | SC-15407 | Rabbit polyclonal |  | 1:100 |  |
| ***p53*** | Santa Cruz, Dallas, Texas, USA | SC-126 | Mouse monoclonal | 1:500 |  |  |
| ***Phospho-Erk1/2*** | Cell Signaling, Danvers, MA, USA | 9101 | Rabbit polyclonal | 1:100 |  |  |
| ***Erk1/2*** | Cell Signaling, Danvers, MA, USA | 9102 | Mouse monoclonal | 1:1000 |  |  |
| ***Phospho-Akt*** | Cell Signaling, Danvers, MA, USA | 9271 | Rabbit polyclonal | 1:500 |  |  |
| ***Akt*** | Cell Signaling, Danvers, MA, USA | 2920 | Mouse monoclonal | 1:500 |  |  |
| ***Phospho-STAT3*** | Cell Signaling, Danvers, MA, USA | 9131S | Rabbit polyclonal | 1:500 |  |  |
| ***STAT3*** | Santa Cruz, Dallas, Texas, USA | SC-8019 | Mouse monoclonal | 1:1000 |  |  |
| ***P21 Waf*** | BD Biosciences, San Jose, CA, USA | 556430 | Mouse monoclonal |  | 1:100 |  |
| ***P21 Waf*** | Cell Signaling, Danvers, MA, USA | 2947S | Rabbit monoclonal | 1:1000 |  |  |
| ***Caspase 3*** | Cell Signaling, Danvers, MA, USA | 9662S | Rabbit polyclonal | 1:500 |  |  |
| ***P63*** | Abcam, Cambridge UK | 63881 | Rabbit polyclonal |  | 1:200 |  |
| ***P63*** | Oncogene,California USA | D10281-9 | Mouse monoclonal | 1:500 |  |  |
| ***P63*** | Atlas, Stockolm Sw | HPA006288 | Mouse monoclonal |  |  | 1:200 |
| ***Vimentin*** | BD Biosciences, San Jose, CA, USA | 550513 | Mouse monoclonal |  |  | 1:200 |
| ***Involucrin*** | Sigma-Aldrich, St. Louis, MO, USA | I9018 | Mouse monoclonal | 1:1000 |  | 1:10000 |
| ***14-3-3 sigma*** | Santa Cruz, Dallas, Texas, USA | SC-100638 | Mouse monoclonal | 1:500 |  | 1:1000 |
| ***α-Smooth Muscle*** | Sigma-Aldrich, St. Louis, MO, USA | A2547 | Mouse monoclonal | 1:1000 |  |  |
| ***Ki-67*** | Cell Signaling, Danvers, MA, USA | 9027 | Rabbit monoclonal |  |  | 1:400 |
| ***GAPDH*** | Santa Cruz, Dallas, Texas, USA | FL-335 | Rabbit polyclonal | 1:1000 |  |  |
| ***HRP-Anti-mouse*** | GE Healthcare UK Limited, Little Chalfont Buckinghamshire | NA931V |  | 1:5000 |  |  |
| ***HRP-Anti-rabbit*** | GE Healthcare UK Limited, Little Chalfont Buckinghamshire | NA934VS |  | 1:5000 |  |  |
| ***Biotinylated-Anti-mouse*** | Vector Laboratories, Inc. Burlingame, CA, USA | BA-2000 |  |  | 1:200 | 1:150 |
| ***Biotinylated-Anti-rabbit*** | Vector Laboratories, Inc. Burlingame, CA, USA | BA-1000 |  |  | 1:200 | 1:150 |
| ***Alexa Fluor™ 555 conjugate Streptavidin*** | Thermo Fischer Scientific, Waltham, MA, USA | S21381 |  |  | 1:500 |  |
| ***Anti-rabbit Alexa Fluor® 488 conjugate*** | Thermo Fischer Scientific, Waltham, MA, USA | A21206 |  |  | 1:200 |  |

| **Supplementary Table 3. Association between marker expression levels and clinicopathologic features of patients with cSCC** | | | | | | | | | | | | | | | | | | | | | | | | | | |
| --- | --- | --- | --- | --- | --- | --- | --- | --- | --- | --- | --- | --- | --- | --- | --- | --- | --- | --- | --- | --- | --- | --- | --- | --- | --- | --- |
|  | **Sex** | |  | **Age, y** | |  | | **Anatomic site** | | | |  | | | **Grade^a^** | | | | | | | | |  | | |
|  | males | females |  | ≤80 | >80 |  | | Head | | Other sites | |  | | | G1 | G2 | | | | | G3 | | |  | | |
| ***Epidermal layers*** | N. (%) | N. (%) | *P*value | N. (%) | N. (%) | *P*value | | N. (%) | | N. (%) | | *P*value |  | N. (%) | | | | N. (%) | | N. (%) | | | *P*value*^b^* | | |  |
| **Trkb** |  |  |  |  |  |  | |  | |  | |  | | |  |  | | | | |  | | |  | | |
| mean (SD) | 130.6 (100.5) | 151.9 (98.8) |  | 151.6 (101.6) | 118.6 (95.8) |  | | 118.0 (98.9) | | 180.2 (89.6) | |  | | | 172.6 (68.8) | 205.6 (96.9) | | | | | 166.9 (100.9) | | |  | | |
| median (IQR) | 112.9 (50.0-232.7) | 155.1 (66.6-250.0) | 0.438 | 169.5 (50.0-250.0) | 100.9 (41.0-177.2) | 0.248 | 100.9 (23.8-194.0) | | 201.9 (115.0-250.1) | | 0.027 | | | 198.9 (140.2-211.4) | | | 246.2 (136.8-280.6) | | 194.0 (80.0-257.0) | | | 0.392 | | |  |  |
| **Yap1nuclear** |  |  |  |  |  |  |  | |  | |  | | |  | | |  | |  | | |  | | |  |  |
| mean (SD) | 172.6 (120.8) | 137.6 (118.8) |  | 160.2 (121.6) | 163.9 (120.9) |  | 159.4 (119.5) | | 167.2 (125.3) | |  | | | 219.3 (99.9) | | | 197.8 (129.6) | | 257.0 (76.4) | | |  | | |  |  |
| median (IQR) | 203.1 (35.4-296.4) | 126.9 (25.6-276.0) | 0.274 | 150.0 (32.5-294.0) | 190.1 (30.5-293.1) | 0.792 | 165.1 (32.5-291.0) | | 200.0 (49.8-298.0) | | 0.709 | | | 245.8 (175.2-298.1) | | | 283.5 (55.6-298.0) | | 294.0 (261.8-297.0) | | | 0.840 | | |  |  |
| **E-cadherin** |  |  |  |  |  |  |  | |  | |  | | |  | | |  | |  | | |  | | |  |  |
| mean (SD) | 83.2 (90.1) | 81.8 (82.9) |  | 91.5 (86.2) | 71.4 (88.9) |  | 77.4 (86.0) | | 94.9 (91.2) | |  | | | 31.7 (28.7) | | | 33.9 (47.2) | | 73.7 (78.2) | | |  | | |  |  |
| median (IQR) | 54.4 (6.0-150.0) | 60.0 (4.0-155.1) | 0.891 | 69.6 (10.0-150.0) | 42.3 (0.0-100.0) | 0.263 | 45.8 (0.0-150.0) | | 60.0 (34.0-155.1) | | 0.318 | | | 26.3 (6.1-55.3) | | | 3.0 (0.0-64.0) | | 60.0 (0.0-161.8) | | | 0.421 | | |  |  |
| **Notch1** |  |  |  |  |  |  |  | |  | |  | | |  | | |  | |  | | |  | | |  |  |
| mean (SD) | 65.3 (84.1) | 85.0 (94.8) |  | 87.0 (98.6) | 51.3 (66.2) |  | 67.4 (82.3) | | 80.4 (99.2) | |  | | | 29.2 (35.1) | | | 33.3 (65.8) | | 9.2 (11.4) | | |  | | |  |  |
| median (IQR) | 32.7 (0.0-100.0) | 49.5 (2.8-150.0) | 0.633 | 52.7 (0.0-150.0) | 25.7 (1.5-56.0) | 0.355 | 32.7 (4.0-100.0) | | 35.4 (0.0-168.3) | | 0.971 | | | 18.2 (1.4-48.1) | | | 3.9 (0.0-37.7) | | 4.0 (0.0-15.4) | | | 0,682 | | |  |  |
|  |  |  |  |  |  |  |  | |  | |  | | |  | | |  | |  | | |  | | |  |  |
| ***Stroma^a^*** |  |  |  |  |  |  |  | |  | |  | | |  | | |  | |  | | |  | | |  |  |
| **Trkb** |  |  |  |  |  |  |  | |  | |  | | |  | | |  | |  | | |  | | |  |  |
| mean (SD) | 0.4 (0.2) | 0.7 (0.1) |  | 0.5 (0.2) | 0.5 (0.2) |  | 0.5 (0.2) | | 0.6 (0.2) | |  | | | 0.6 (0.1) | | | 0.5 (0.2) | | 0.4 (0.2) | | |  | | |  |  |
| median (IQR) | 0.5 (0.3-0.6) | 0.6 (0.6-0.8) | 0.005 | 0.6 (0.4-0.6) | 0.5 (0.4-0.6) | 0.771 | 0.5 (0.4-0.6) | | 0.06 (0.6-0.7) | | 0.09 | | | 0.6 (0.5-0.7) | | | 0.6 (0.3-0.6) | | 0.4 (0.3-0.6) | | | 0.237 | | |  |  |
| **Yap1nuclear** |  |  |  |  |  |  |  | |  | |  | | |  | | |  | |  | | |  | | |  |  |
| mean (SD) | 0.8 (0.2) | 0.7 (0.2) |  | 0.8 (0.2) | 0.8 (0.2) |  | 0.8 (0.2) | | 0.8 (0.1) | |  | | | 0.8 (0.2) | | | 0.7 (0.2) | | 0.8 (0.1) | | |  | | |  |  |
| median (IQR) | 0.8 (0.6-0.9) | 0.8 (0.6-0.8) | 0.58 | 0.8 (0.6-0.9) | 0.8 (0.6-0.9) | 0.923 | 0.7 (0.6-0.9) | | 0.8 (0.8-0.8) | | 0.472 | | | 0.8 (0.6-0.9) | | | 0.7 (0.6-0.8) | | 0.8 (0.7-0.9) | | | 0.548 | | |  |  |
| **Vimentin** |  |  |  |  |  |  |  | |  | |  | | |  | | |  | |  | | |  | | |  |  |
| mean (SD) | 1.7 (0.3) | 1.8 (0.5) |  | 1.7 (0.4) | 1.8 (0.4) |  | 1.7 (0.4) | | 1.9 (0.4) | |  | | | 2.0 (0.3) | | | 1.8 (0.4) | | 1.5 (0.3) | | |  | | |  |  |
| median (IQR) | 1.8 (1.5-2.0) | 1.7 (1.4-2.3) | 0.761 | 1.7 (1.4-2.0) | 1.8 (1.5-2.0) | 0.778 | 1.6 (1.4-2.0) | | 1.8 (1.7-2.2) | | 0.205 | | | 1.9 (1.7-2.2) | | | 1.7 (1.5-2.1) | | 1.4 (1.2-1.8) | | | 0.013^c^ | | |  |  |
| **Notch1** |  |  |  |  |  |  |  | |  | |  | | |  | | |  | |  | | |  | | |  |  |
| mean (SD) | 0.5 (0.3) | 0.6 (0.2) |  | 0.5 (0.3) | 0.6 (0.2) |  | 0.6 (0.3) | | 0.5 (0.2) | |  | | | 0.6 (0.1) | | | 0.4 (0.3) | | 0.7 (0.2) | | |  | | |  |  |
| median (IQR) | 0.5 (0.5-0.7) | 0.7 (0.5-0.8) | 0.319 | 0.5 (0.2-0.6) | 0.7 (0.5-0.7) | 0.133 | 0.7 (0.5-0.8) | | 0.5 (0.5-0.6) | | 0.643 | | | 0.7 (0.5-0.7) | | | 0.5 (0.2-0.6) | | 0.7 (0.5-0.8) | | | 0.196 | | |  |  |
| *Abbreviation*: SCC, Squamous Cell Carcinoma; SD, Standard Deviation; IQR, Interquartile Range. | | | | | | | | | | | |  | | |  |  | | | | |  | | |  | | |
| a: only for invasive SCC | |  |  |  |  |  | |  | |  | |  | | |  |  | | | | |  | | |  | | |
| b: Mann-Whitney U test | |  |  |  |  |  | |  | |  | |  | | |  |  | | | | |  | | |  | | |
| c: Cuzick’s test for trend across the three categories | | |  |  |  |  | |  | |  | |  | | |  |  | | | | |  | | |  | | |

|  |
| --- |

**Supplementary Table 4. Association between marker expression levels and stage/tumor thickness of invasive SCC**

|  | **Stage** | |  | **Tumor thickness, mm** | |  | |
| --- | --- | --- | --- | --- | --- | --- | --- |
|  | pT1 | pT2 |  | ≤2 | >2 |  | |
| ***Epidermal layers*** |  |  | *P*value |  |  | *P*value | |
| **Trkb** |  |  |  |  |  |  | |
| mean (SD) | 176.29 (98.35) | 210.36 (72.28) |  | 187.97 (100.12) | 179.22 (84.80) |  | |
| median (IQR) | 196.00 [98.70–262.00] | 222.94 [167.37–255.70] | 0,514 | 220.00 [114.68–262.00] | 176.25 [119.62–255.70] | 0,803 |  |
| **Yap1nuclear** |  |  |  |  |  |  |  |
| mean (SD) | 193.55 (118.86) | 275.45 (36.96) |  | 226.82 (106.99) | 192.78 (117.62) |  |  |
| median (IQR) | 262.00 [83.31–296.64] | 295.18 [269.82–296.84] | 0,308 | 291.00 [175.18–298.06] | 233.66 [109.15–289.50] | 0,183 |  |
| **E-cadherin** |  |  |  |  |  |  |  |
| mean (SD) | 39.15 (48.89) | 48.99 (78.17) |  | 33.70 (46.92) | 53.23 (67.42) |  |  |
| median (IQR) | 19.27 [0.00–59.13] | 5.00 [0.00–73.25] | 0,82 | 8.20 [0.00–54.42] | 21.67 [0.00–88.17] | 0,712 |  |
| **Notch1** |  |  |  |  |  |  |  |
| mean (SD) | 34.00 (55.09) | 8.08 (11.94) |  | 17.69 (23.43) | 42.93 (72.18) |  |  |
| median (IQR) | 15.45 [0.00–45.09] | 4.00 [1.00–7.75] | 0,379 | 3.00 [0.00–38.00] | 13.84 [4.00–32.87] | 0,32 |  |
|  |  |  |  |  |  |  |  |
| ***Stroma^a^*** |  |  |  |  |  |  |  |
| **Trkb** |  |  |  |  |  |  |  |
| mean (SD) | 0.48 (0.21) | 0.51 (0.15) |  | 0.46 (0.20) | 0.53 (0.19) |  |  |
| median (IQR) | 0.53 [0.35–0.59] | 0.57 [0.46–0.60] | 0,642 | 0.53 [0.35–0.58] | 0.56 [0.45–0.61] | 0,421 |  |
| **Yap1nuclear** |  |  |  |  |  |  |  |
| mean (SD) | 0.75 (0.20) | 0.76 (0.19) |  | 0.80 (0.18) | 0.68 (0.19) |  |  |
| median (IQR) | 0.77 [0.60–0.83] | 0.78 [0.62–0.90] | 0,78 | 0.78 [0.71–0.88] | 0.62 [0.52–0.81] | 0,174 |  |
| **Vimentin** |  |  |  |  |  |  |  |
| mean (SD) | 1.78 (0.40) | 1.63 (0.34) |  | 1.73 (0.41) | 1.75 (0.36) |  |  |
| median (IQR) | 1.79 [1.47–2.15] | 1.66 [1.33–1.94] | 0,537 | 1.58 [1.47–2.09] | 1.84 [1.43–1.97] | 0,905 |  |
| **Notch1** |  |  |  |  |  |  |  |
| mean (SD) | 0.51 (0.26) | 0.67 (0.17) |  | 0.55 (0.29) | 0.55 (0.19) |  |  |
| median (IQR) | 0.54 [0.39–0.69] | 0.73 [0.53–0.77] | 0,221 | 0.55 [0.47–0.75] | 0.53 [0.46–0.72] | 0,76 |  |
| Mann-Whitney U test | |  |  |  |  |  | |
|  | |  |  |  |  |  | |
|  | | |  |  |  |  | |

| **Supplementary Table 5. Characteristics of SCC cell lines.** | | |  |
| --- | --- | --- | --- |
|  | Cell lines | |  |
|  | SCC13 | SCC15 | A431 |
| **Gender***^$^ | Female | Male | Female |
| **Age**, years*^$^ | 56 | 55 | 85 |
| **Site***^$^ | face | tongue | skin |
| **Therapy*** | radiotherapy | none |  |
| **Colony Forming Efficiency** (%)*/*** | 0.02 -3.5 | 0.2 |  |
| **Involucrin expression (vs ctrl)***** | 0.01 | 0.01 |  |
| **TP53 gene^#,##,^**^$$^ | G → A | +ATCTG | c.818G > A |
| **p53 expression^#,##,^**^$$^ | +++ | - | +++ |
| **Irreversible growth arrest**** | ++ | ++ |  |
| **Terminal differentiation**** | +/- | ++ |  |
| **Colony Forming Efficiency (%) in suspension microenvironment**** | 30 | 20 |  |
| For SCC13 and SCC15:  *Rheinwald and Beckett, 1981 (Cancer research) | | |  |
| **Scott et al., 1988 (Am J Pathol.) | | |  |
| ***Jensen et al., 2008 (Cancer Lett.) | | |  |
| #Burns et al., 1993 (Br J Cancer) |  |  |  |
| ##Yeudal et al., 1997 (Mol Carcinog) |  |  |  |

For A431:

^$^Giard DJ, et al. 1973 (J Natl Cancer Inst.)

^$$^Wang P. et al. 2025 (Genome Biology)

| **Supplementary Table 6. Characteristics of patients from whom the CAFs were isolated.** | | |  |
| --- | --- | --- | --- |
|  | Primary cell lines | | |
|  | CAF1 | CAF2 | CAF3 |
| **Gender** | Male | Female | Male |
| **Age**, years | 64 | 87 | 74 |
| **Site** | palm | face | palm |
|  | | |  |
|  | | |  |
|  | | |  |
|  |  |  |  |
|  |  |  |  |
