# Supplementary material for: TRKB-based signature identifies high-risk squamous cell carcinoma cases and TRKB blockade reprograms tumor and stromal cells toward suppressive phenotypes
Source: J Biomed Sci. 2026 Feb 25;33:22. doi: 10.1186/s12929-026-01227-0 (PMC12934051; doi:10.1186/s12929-026-01227-0)
Supplement: Supplementary file 2 — Additional file2 (DOCX 2697 KB) [file 12929_2026_1227_MOESM2_ESM.docx]

**Supplementary Figures and Legends**

**
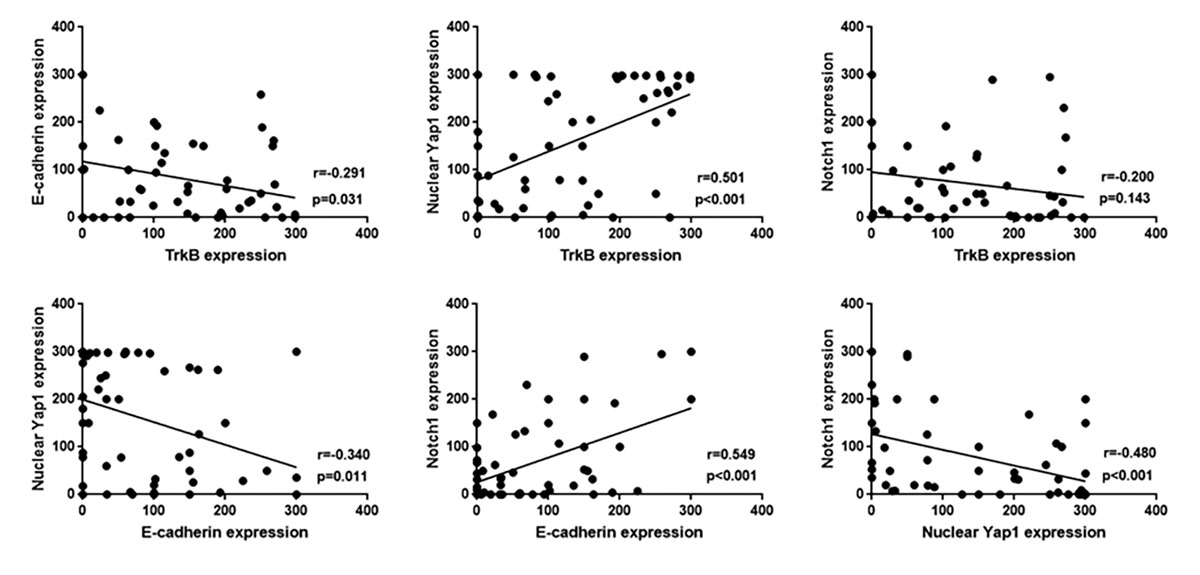
**

**Supplementary Figure 1. Correlations between TrkB, E-cadherin, Yap1, and Notch1.** Linear regression analyses are shown for each data series, with the *r* coefficient and the two-tailed *p* value.


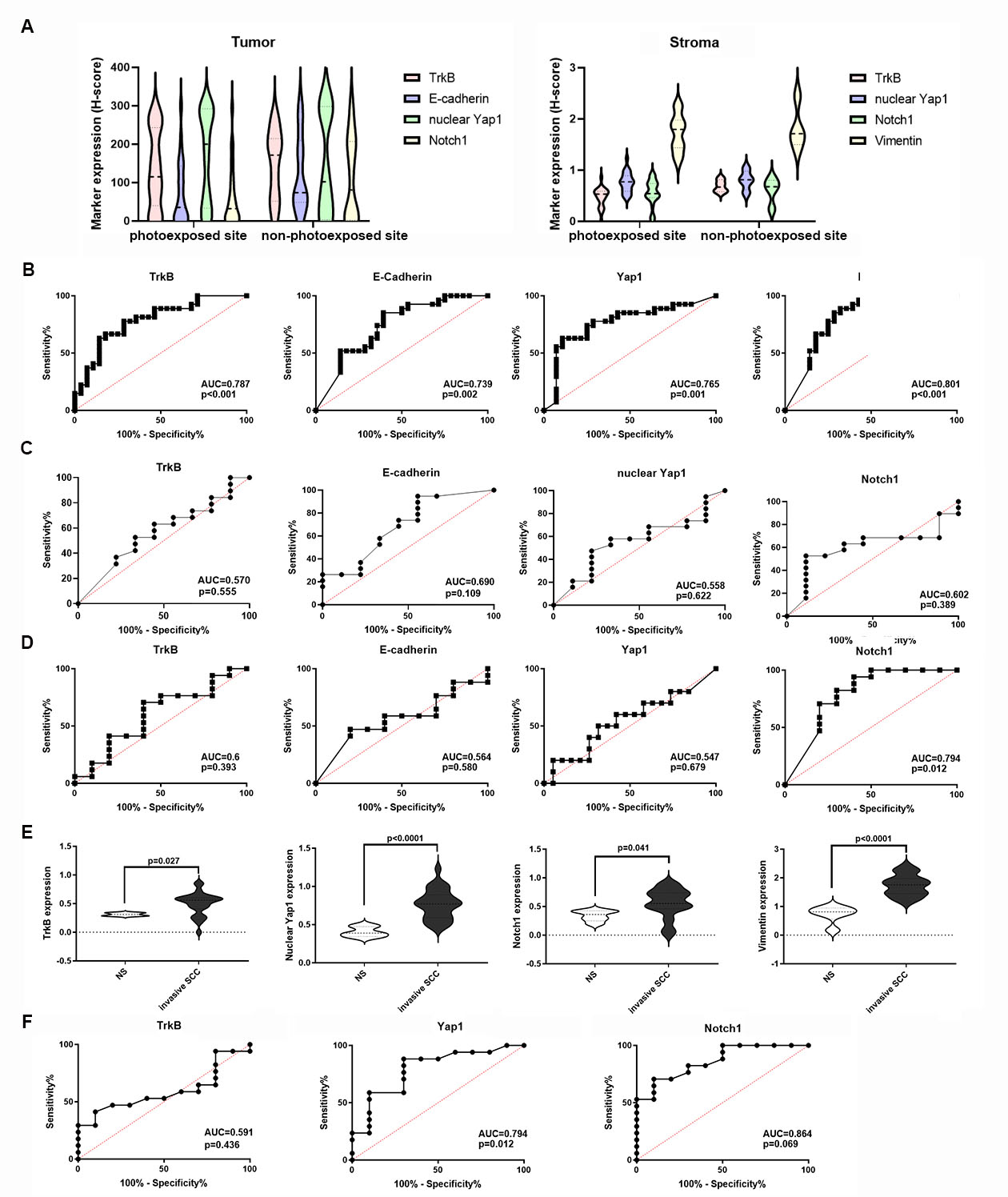


**Supplementary Figure 2. Assessment of the diagnostic power of single biomarkers.** Differential expression of TrkB, E-cadherin or vimentin, Yap1, and Notch1 in keratinocytes or stromal cells of SCCs from photoesposed or non-photoexposed sites (A). ROC analysis for discriminating in situ vs invasive cSCCs (B), low- and high-risk in situ cSCCs (C), and invasive cSCCs (D) using epithelial values of each protein. (E) Differential expression of TrkB, Yap1, and Notch1 in the dermis of normal skin (NS) vs invasive cSCCs. Data are shown as mean ± SD. Significance was determined using a two-tailed Student’s t-test. Significance is indicated in the figure. (F) ROC analysis for discriminating low- and high-risk cSCCs using stromal value of each protein.

**
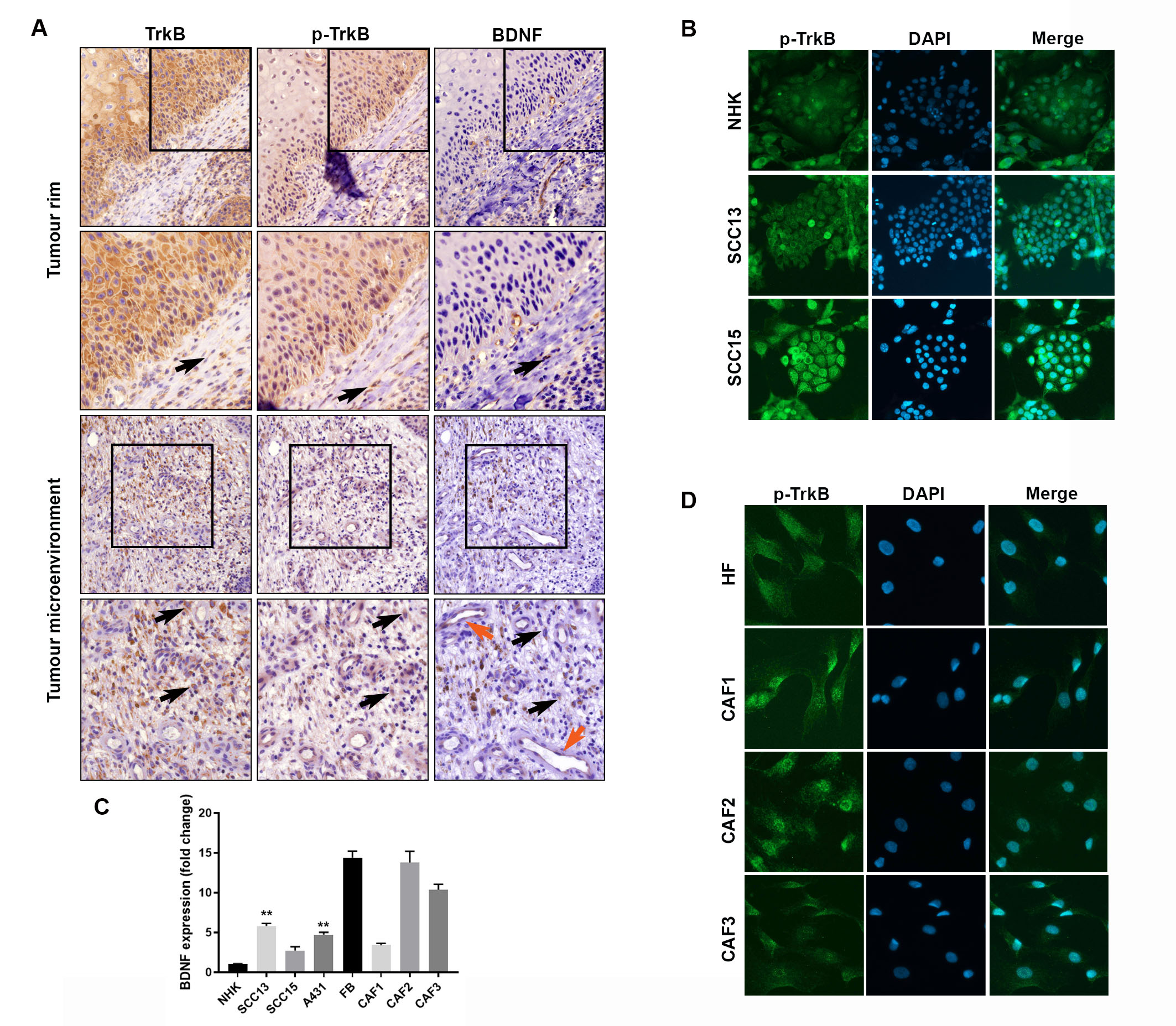
**

**Supplementary Figure 3. Phospho TrkB (p-TrkB) and BDNF expression.** (A) Representative images of paraffin-embedded specimens of invasive SCC immunostained with antibodies against TrkB, p-TrkB and BDNF (20x magnification; 40x magnification for insets). (B) Representative images of p-TrkB immunostaining (green) in primary normal human keratinocytes (NHK) and SCC cells. DAPI (blue) was used for nuclear staining. (C) Relative BDNF expression in NHK, SCC cells, fibroblasts (FB) and CAFs by RTqPCR. (D) Representative images of p-TrkB immunostaining (green) in FB and CAFs. DAPI (blue) was used for nuclear staining. All immunofluorescence images were acquired at 20x magnification.

**
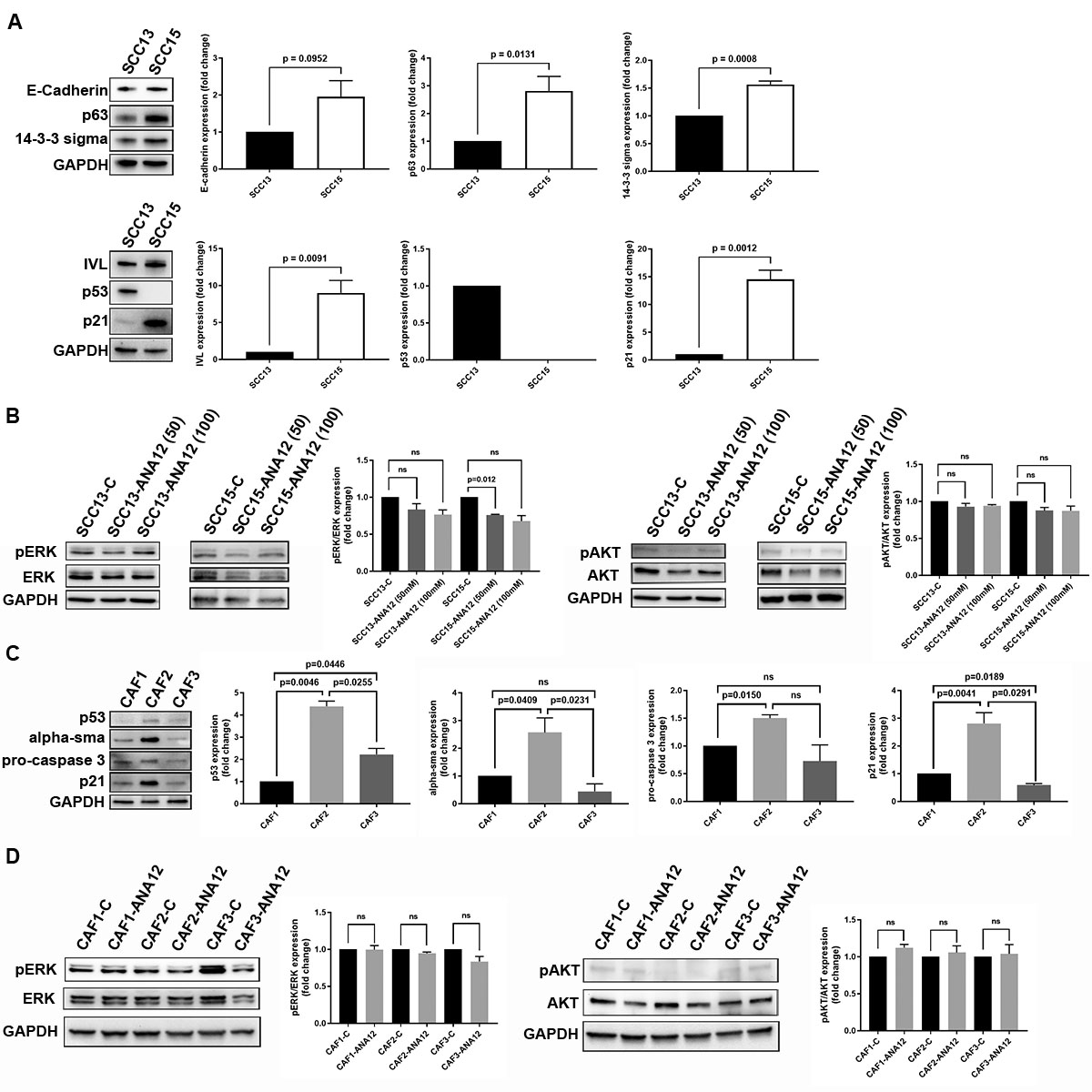
**

**Supplementary Figure 4. Basal levels of protein expression and ANA-12 modulation of ERK and AKT pathways.** Representative immunoblots and related densitometric values (n = 3) of E-cadherin, p63, 14-3-3σ, involucrin (IVL), p53, and p21 in SCC13 and SCC15 (A), of pERK, ERK, pAKT, and AKT in untreated vs ANA-12-treated SCC cells, of α-SMA, pro-caspase 3, and p21 in CAF1-3, of pERK, ERK, pAKT, and AKT in untreated vs ANA-12-treated CAFs. GAPDH was the loading control. All data are shown as mean ± SD. Significance was determined using a two-tailed Student’s t-test. Significance is indicated in the figure. ns = not significant.

**
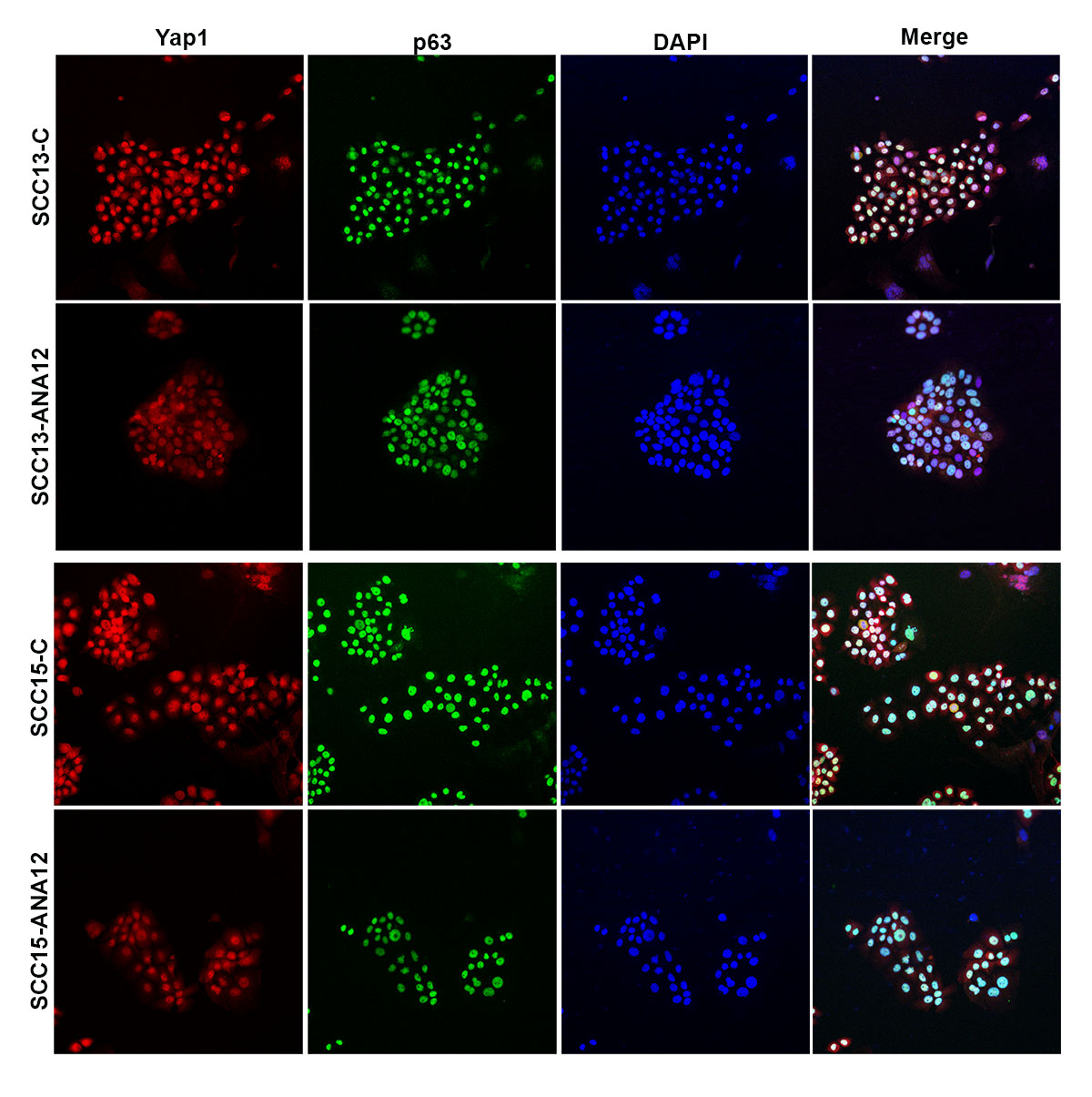
**

**Supplementary Figure 5. Co-expression of Yap1 and p63.** Representative images of Yap1 (red) and p63 (green) immunostaining in untreated vs ANA-12-treated SCC cells. DAPI (blue) was used for nuclear staining. Images were acquired at 20x magnification.

**
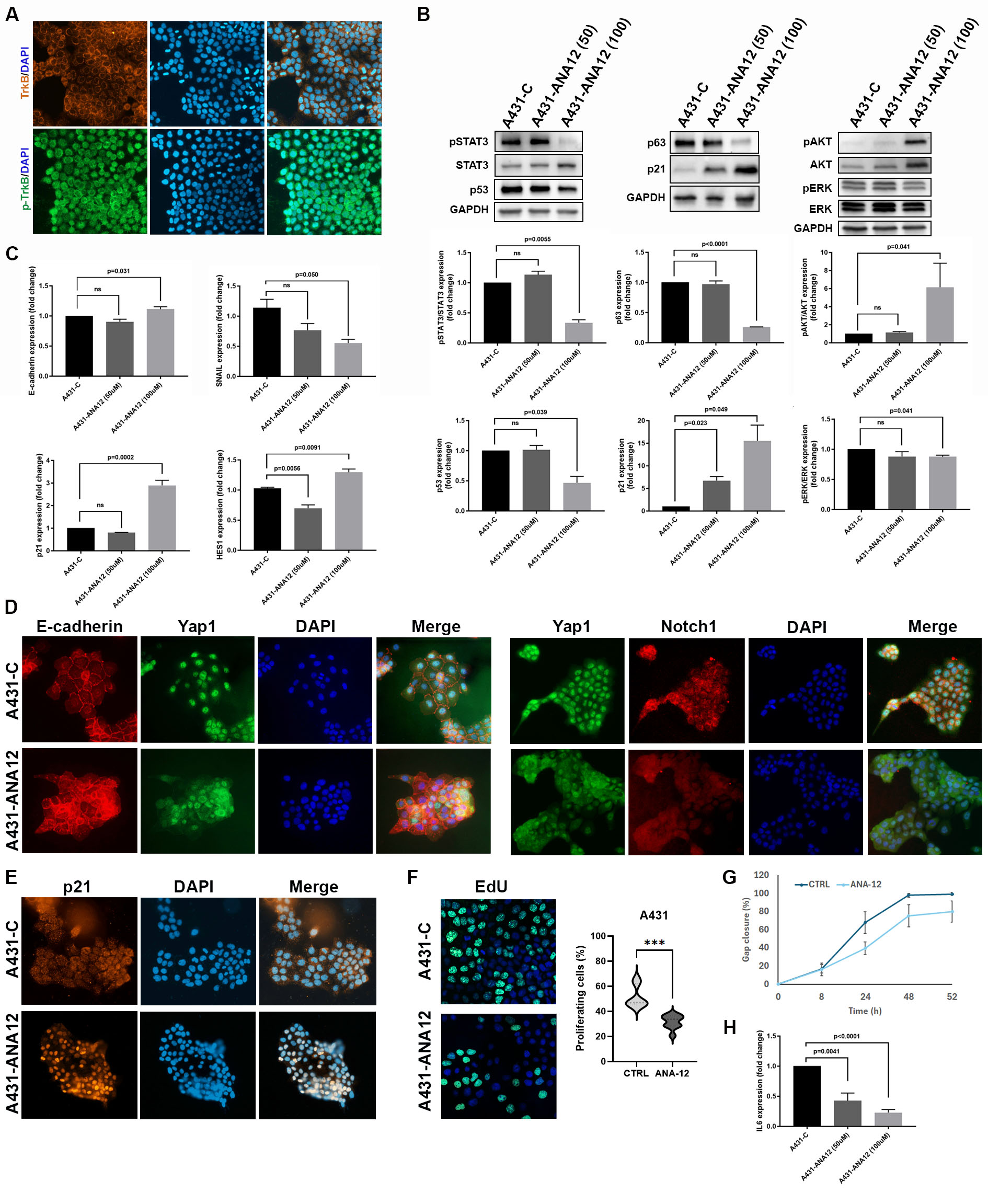
**

**Supplementary Figure 6. Validation of key signaling on A431 cells.** (A) Representative images of p-TrkB (orange) and p-TrkB (green) immunostaining in A431cells. DAPI (blue) was used for nuclear staining. (B) Representative immunoblots and related densitometric values of pSTAT3, STAT3, p53, p63, p21, pERK, ERK, pAKT and AKT in untreated vs ANA-12-treated A431. GAPDH was the loading control. (C) Fold-change expression of Snail, E-cadherin, p21 and Hes1 in untreated vs ANA-12-treated A431 cells by RTqPCR. (D) Representative images of E-cadherin (red) and Yap1 (green) immunostaining in untreated vs ANA-12-treated A431 cells, and Yap1 (green) and Notch1 (red) immunostaining in untreated vs ANA-12-treated A431 cells. (E) Representative images of p21 (orange) immunostaining in untreated vs ANA-12-treated A431 cells. (F) Representative images of proliferating (green) and total (blue) cells in untreated vs ANA-12-treated A431 cells and percentage values. (G) Values of gap closure of A431 cells against time after treatments. (H) Fold-change expression of IL6 in untreated vs ANA-12-treated A431 cells by RTqPCR. All data are shown as mean ± SD. Significance was determined using a two-tailed Student’s t-test and is indicated in the figure. ns = not significant. Images were acquired at 20x magnification.

**
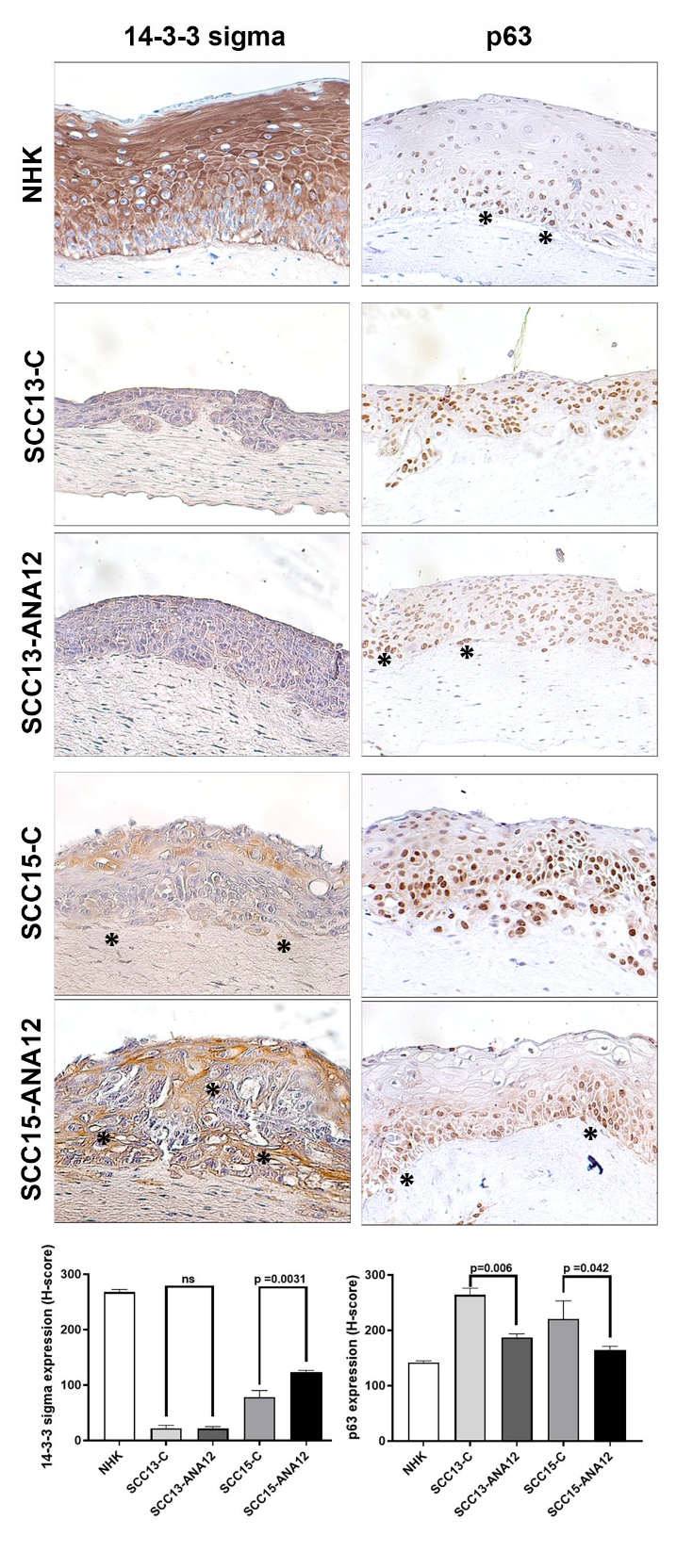
**

**Supplementary Figure 7. Immunostaining of 3D models.** Representative images of paraffin-embedded specimens of NHK and untreated vs ANA-12-treated SCC 3D models immunostained with antibodies against 14-3-3σ and p63. The histological index (H-index) of each specimen was calculated. Data are shown as mean ± SD. Significance was determined using a two-tailed Student’s t-test. Significance is indicated in the figure. ns = not significant. Images were acquired at 20x magnification.
