# Supplementary material for: TRKB-based signature identifies high-risk squamous cell carcinoma cases and TRKB blockade reprograms tumor and stromal cells toward suppressive phenotypes
Source: J Biomed Sci. 2026 Feb 25;33:22. doi: 10.1186/s12929-026-01227-0 (PMC12934051; doi:10.1186/s12929-026-01227-0)

Figure 3D (SCC13)

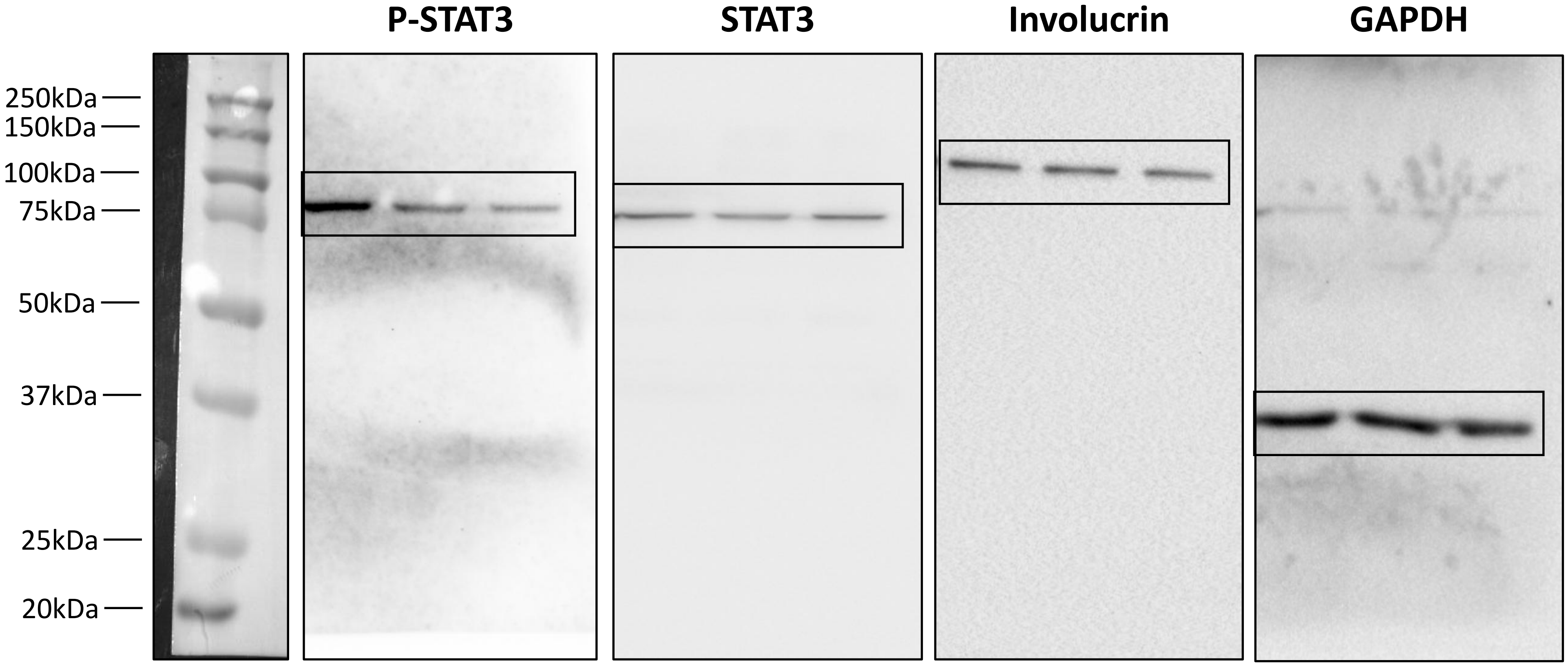

Figure 3D (SCC13)

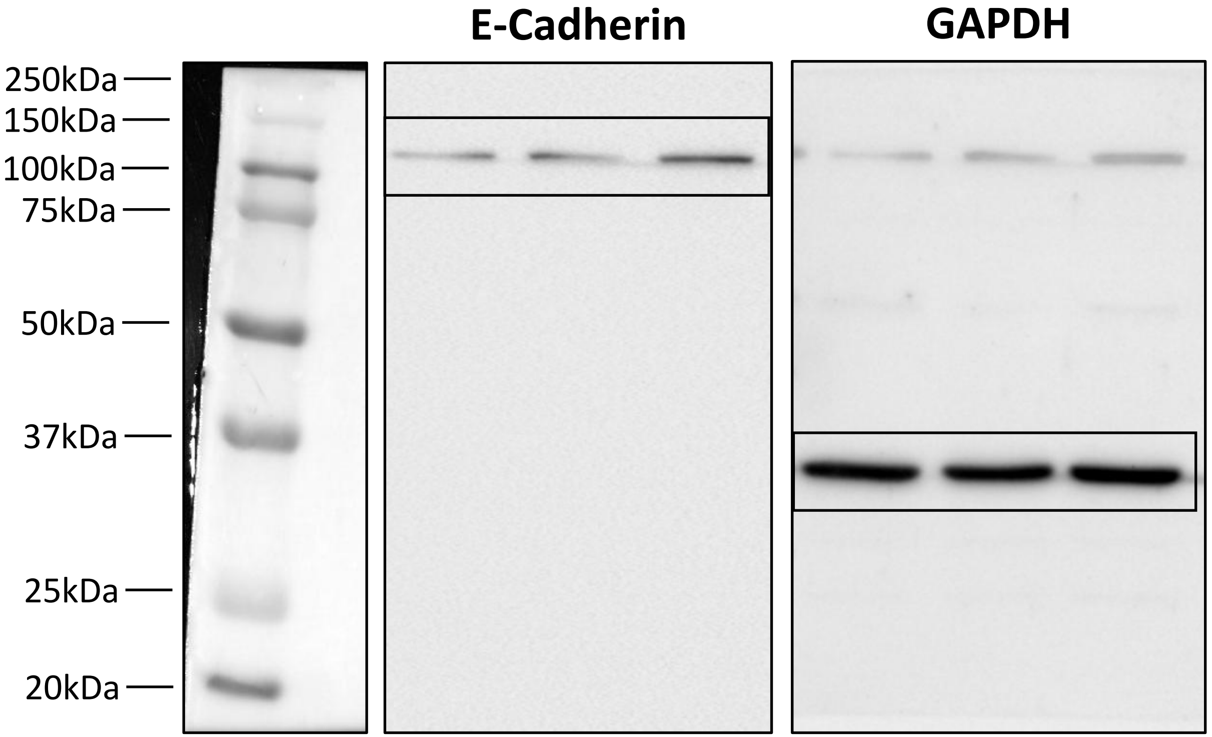

Figure 3D (SCC13)

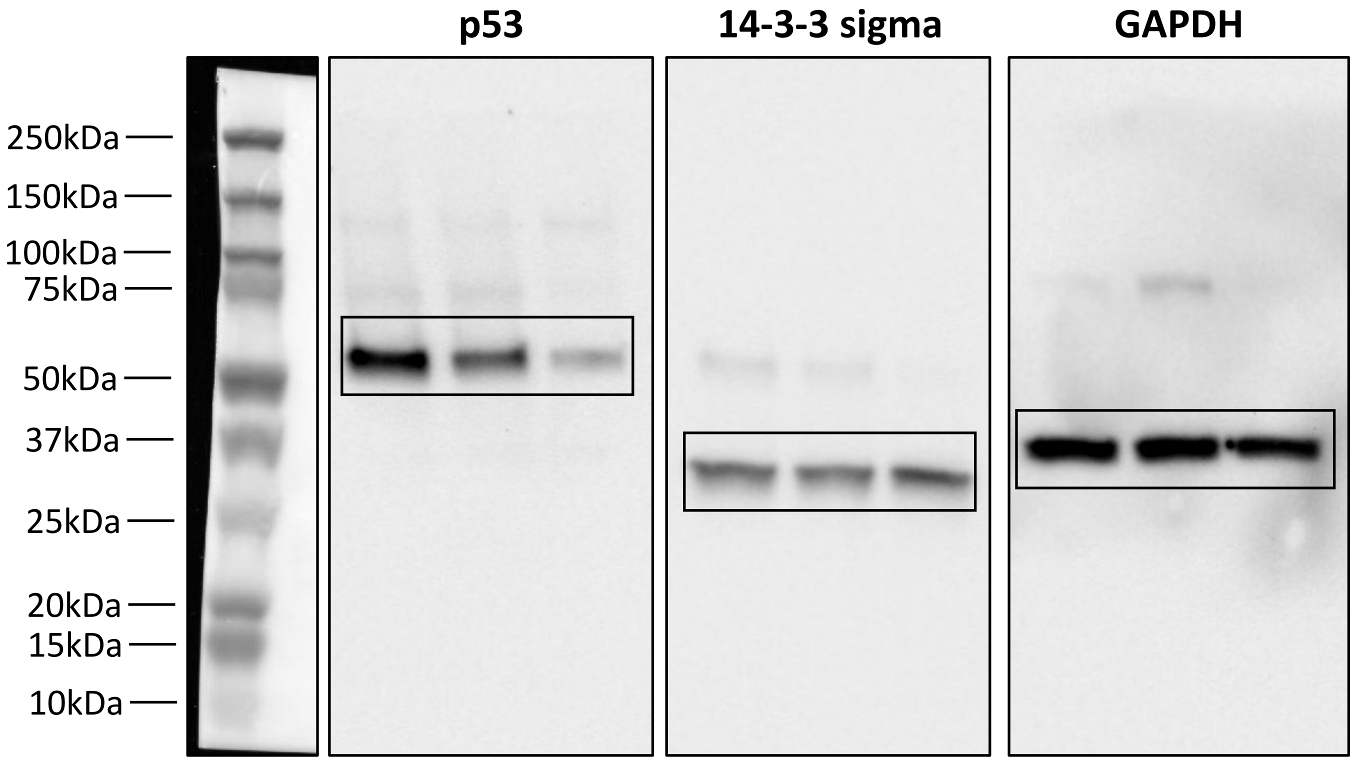

Figure 3D (SCC13)

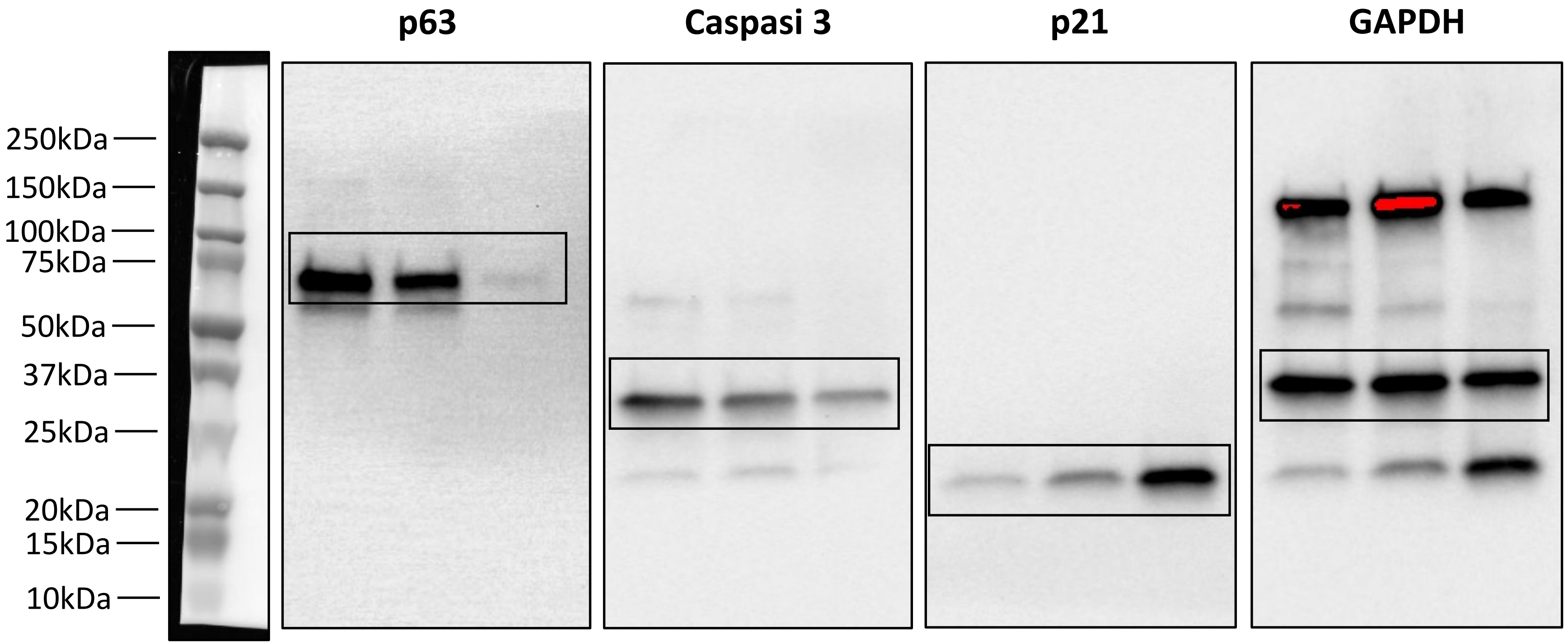

Figure 3D (SCC15)

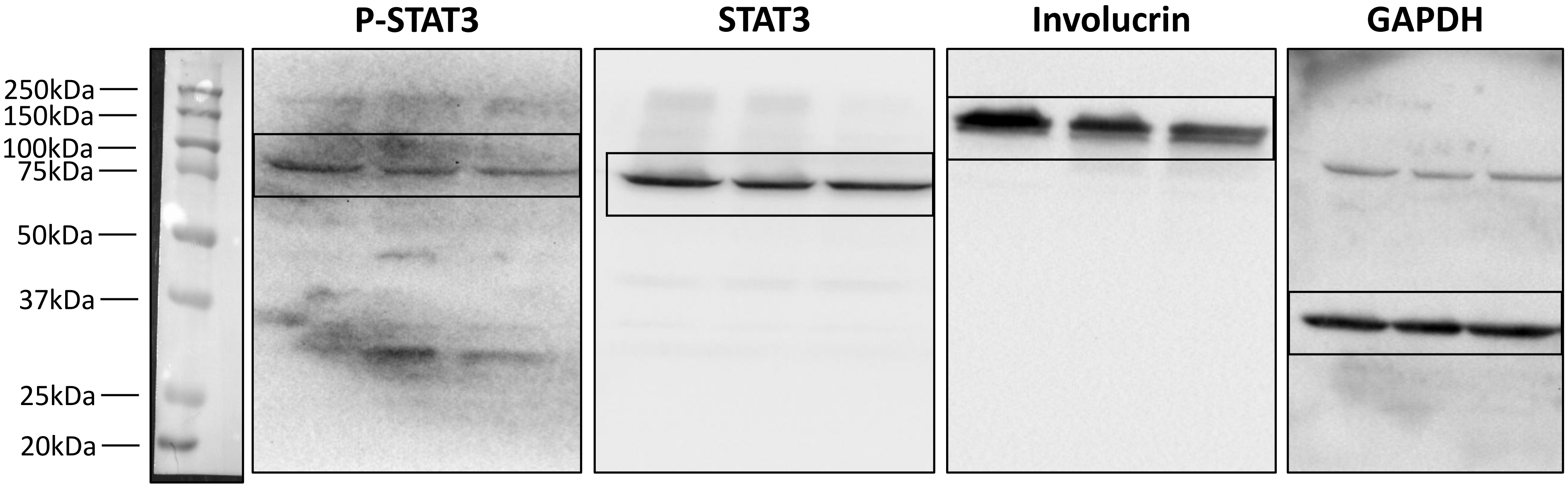

Figure 3D (SCC15)

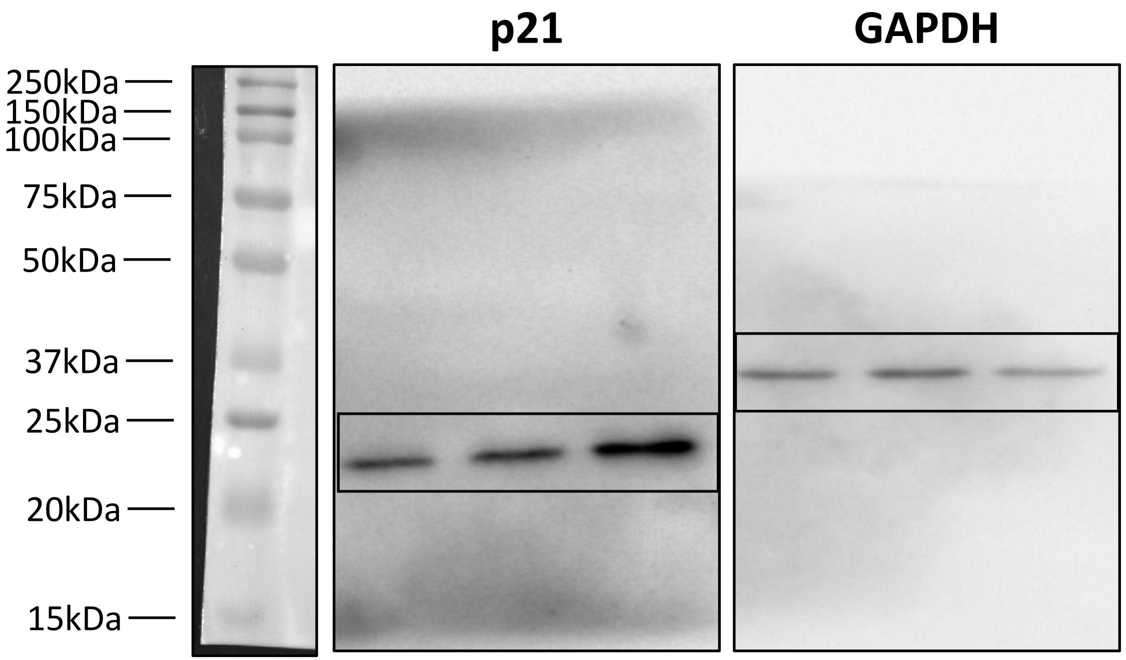

Figure 3D (SCC15)

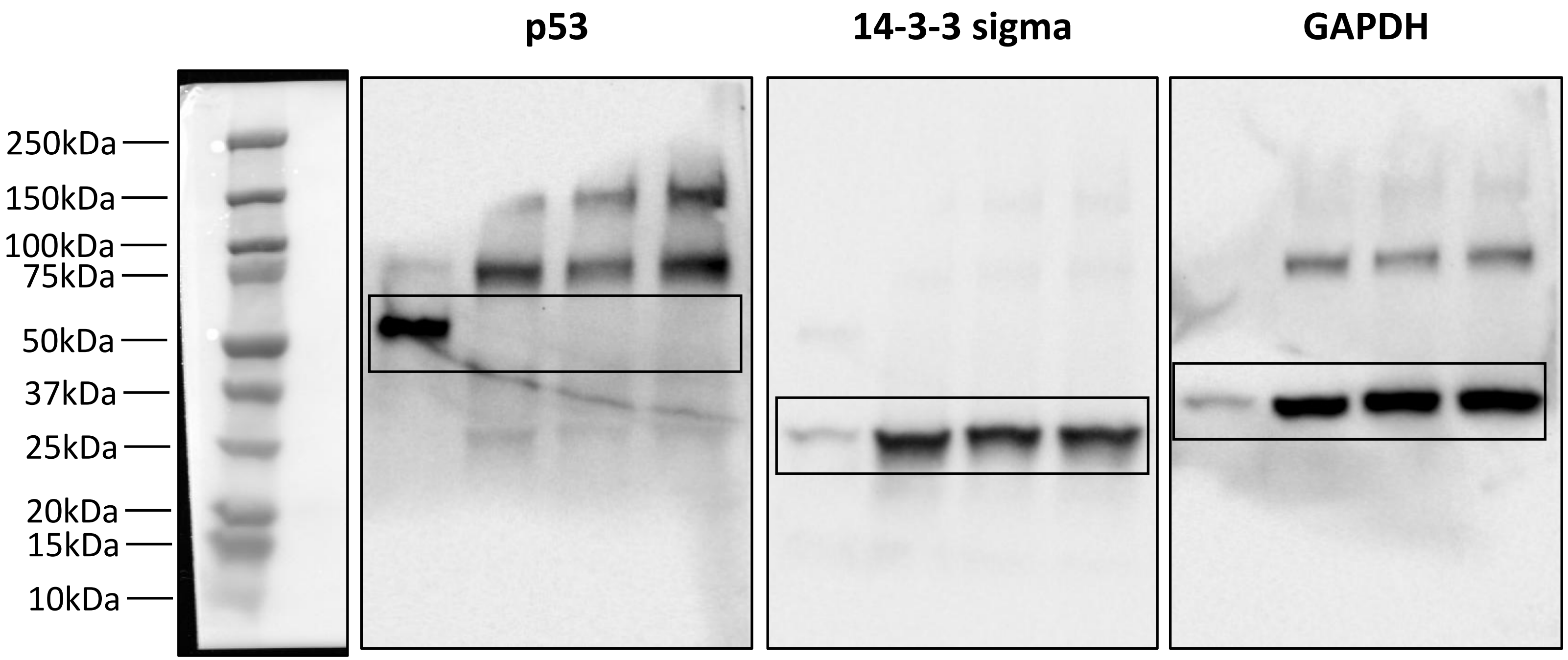

Figure 3D (SCC15)

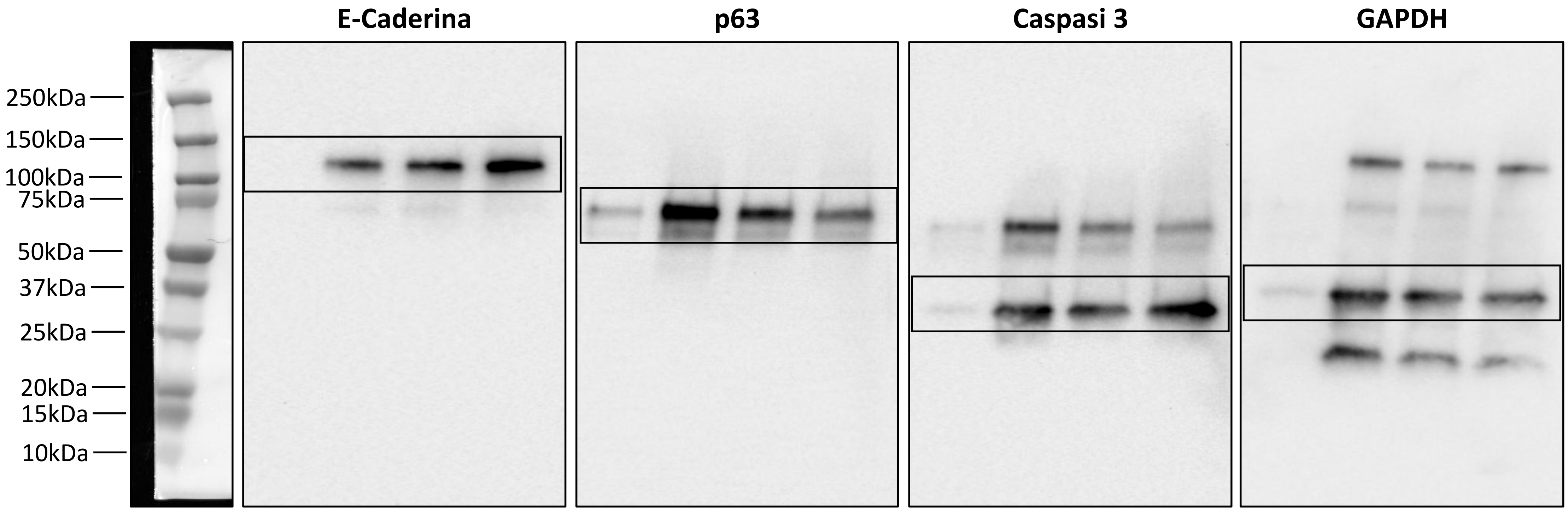

Figure 5C (CAF1 & CAF2)

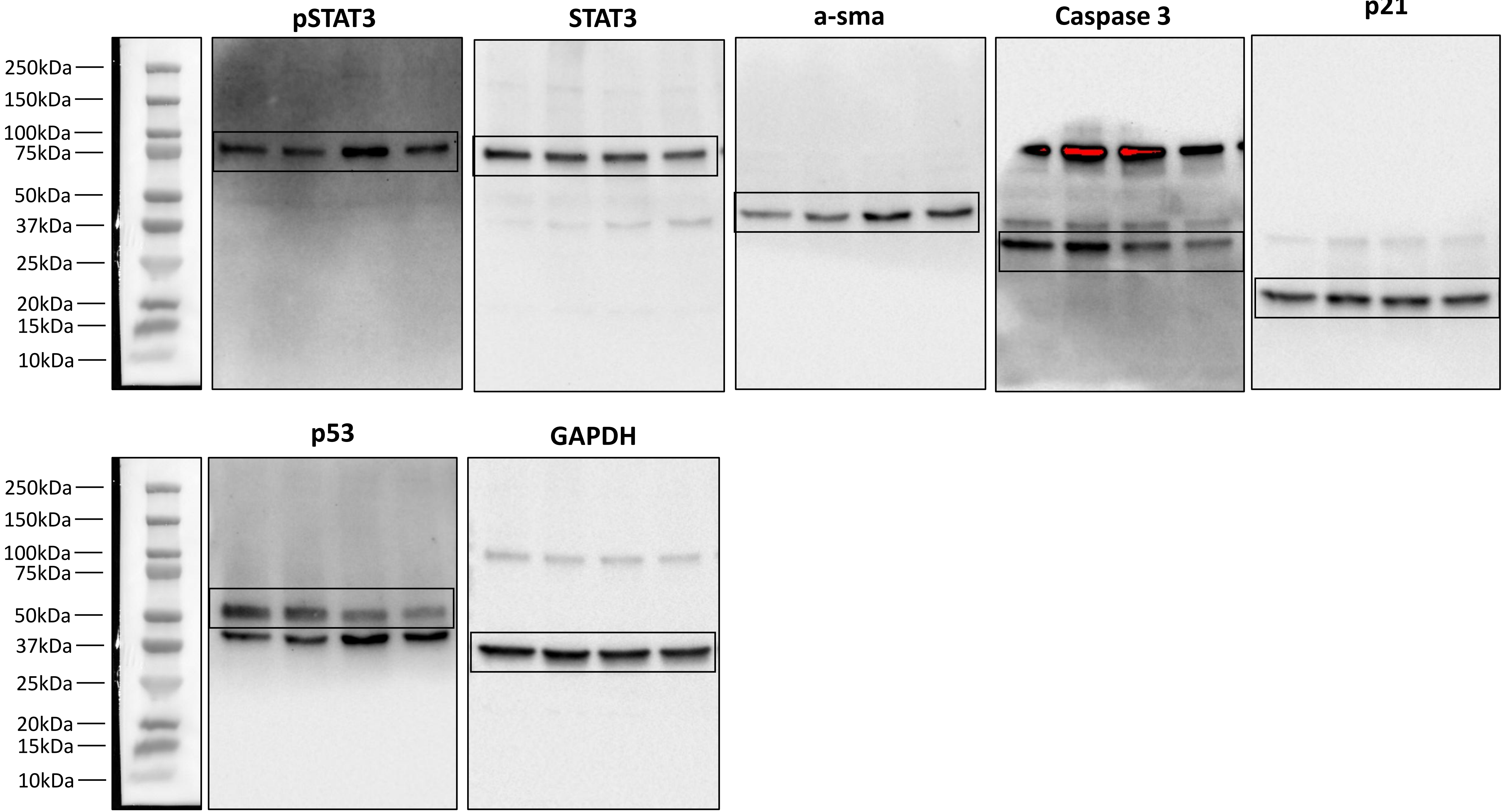

Figure 5C (CAF3)

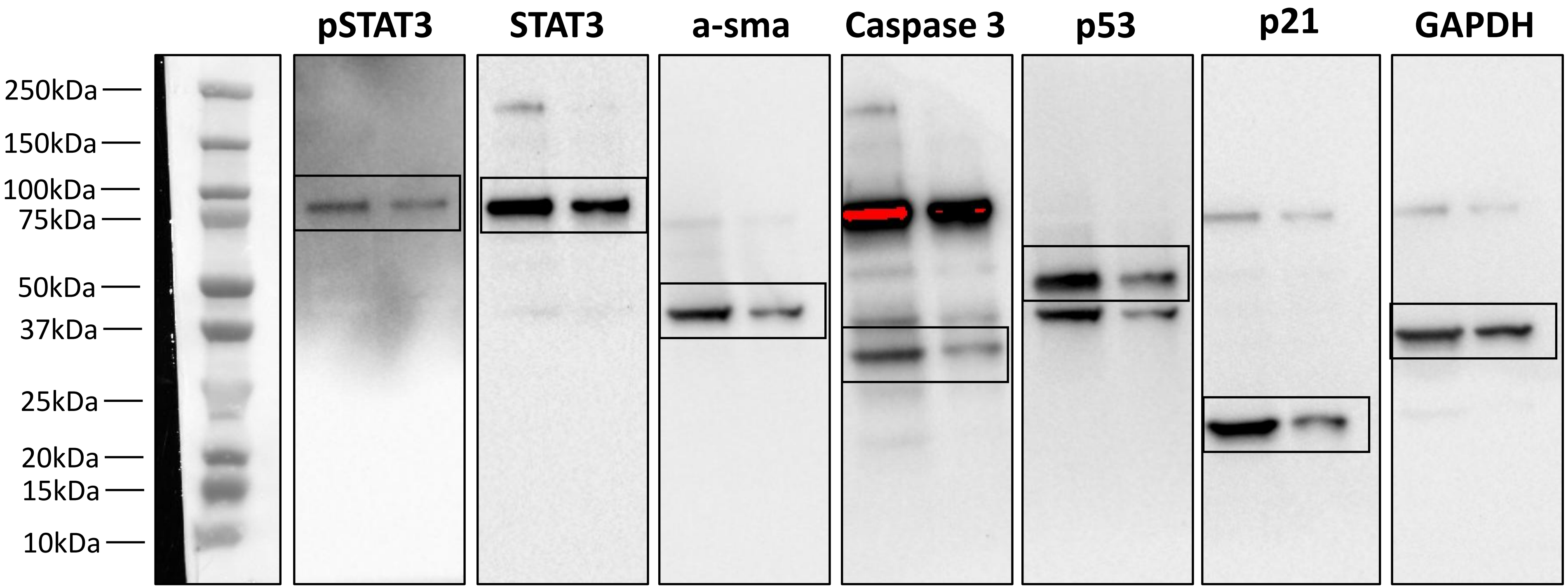

Supplement: Supplementary file 3 — Additional file3 (PDF 777 KB) [file 12929_2026_1227_MOESM3_ESM.pdf]
